# Decoupled Climatic Drivers of Tree and Ground‐Layer Carbon Uptake in Mountain Ecosystems Around the World

**DOI:** 10.1111/gcb.70877

**Published:** 2026-04-17

**Authors:** Max Mallen‐Cooper, Maja K. Sundqvist, David A. Wardle, Radim Šarlej, Rose E. Brinkhoff, Aimée T. Classen, Eliška Kuťáková, Daniel B. Metcalfe, M. Noelia Barrios‐Garcia, Julie R. Deslippe, Kobayashi Makoto, Jane Mallen‐Cooper, Barryette Oberholzer, Juan Paritsis, Jérémy Puissant, Mariano A. Rodriguez‐Cabal, Kohsuke Tanigawa, Susanna E. Venn, Paul Kardol

**Affiliations:** ^1^ Department of Forest Ecology and Management Swedish University of Agricultural Sciences Umeå Sweden; ^2^ Department of Ecology, Environment and Geoscience Umeå University Umeå Sweden; ^3^ Centre for Environmental and Climate Science Lund University Lund Sweden; ^4^ Department of Ecology and Evolutionary Biology University of Michigan Ann Arbor Michigan USA; ^5^ The Rocky Mountain Biological Laboratory Crested Butte Colorado USA; ^6^ CENAC‐APN, CONICET Bariloche Argentina; ^7^ Centre for Biodiversity and Restoration Ecology, School of Biological Sciences Victoria University of Wellington Wellington New Zealand; ^8^ Uryu Experimental Forest, Field Science Center for Northern Biosphere Horonobe Japan; ^9^ 8 Tudor Pl St Ives Chase New South Wales Australia; ^10^ INIBIOMA CONICET–Universidad Nacional del Comahue Bariloche Argentina; ^11^ Laboratoire d'Ecologie Alpine (LECA) Université Grenoble Alpes, Université Savoie Mont Blanc, CNRS Grenoble France; ^12^ Grupo de Ecología de Invasiones, INIBIOMA CONICET–Universidad Nacional del Comahue Bariloche Argentina; ^13^ School of Life and Environmental Sciences Deakin University Melbourne Victoria Australia; ^14^ Department of Forest Mycology and Plant Pathology Swedish University of Agricultural Sciences Uppsala Sweden

**Keywords:** alpine, arctic, carbon cycling, carbon flux, flux chamber, range shift, space‐for‐time, species turnover, subalpine, subarctic

## Abstract

One of the key ecological processes affected by climate change is plant carbon uptake. However, there is substantial uncertainty about how plant carbon uptake will respond to warming in mountain ecosystems, which are known for sharp temperature gradients and abrupt shifts in vegetation structure. Specifically, we lack an understanding of whether these response trajectories over time will be linear or non‐linear, and how they might vary among mountain ecosystems globally. Here, we measured ecosystem Gross Primary Productivity (GPP) along forest‐tundra elevational gradients in the mountain regions of five countries (Argentina, Australia, France, Sweden, USA) to infer future trajectories of carbon uptake, and whether any non‐linear changes might occur. We also examined the role of microclimate in driving GPP responses. We found that whole‐ecosystem GPP increased with increasing macroclimatic temperature (decreasing elevation), but this response was dominated by a sharp non‐linear increase at the transition from tundra to forest (i.e., the treeline). In contrast, ground‐layer GPP was largely independent of macroclimate, but often responded strongly, and linearly, to microclimate (growing degree days > 5°C, mean growing season temperature). This pattern reflected a frequent decoupling of microclimate from the expected temperature‐elevation relationship, likely driven by such processes as cold‐air drainage, limited near‐surface air mixing, and shading by trees. The contrasting responses of GPP among global temperature gradients indicates strong context dependence at both local and continental scales, although in a few cases, biomass and leaf nitrogen were important moderators. These findings suggest that future shifts in carbon uptake in mountains will be mainly controlled by tree range expansion. Our results highlight the need to consider species responses on different spatial scales, and to increase representation of undersampled regions to capture the full breadth of ecological responses to climate change.

## Graphical abstract

Across four continents, we investigated how temperature controls the productivity of high mountain vegetation. We found that the productivity of plants growing close to the ground (< 1 m) tends to be controlled by highly localised “microclimate” temperatures, while the productivity of trees was strongly linked to broader‐scale temperatures. Our findings suggest that, if trees are able to spread up high mountain slopes as the world warms, it will probably lead to a major increase in productivity in these environments, although this productivity could be balanced out by the carbon dioxide that the trees respire.

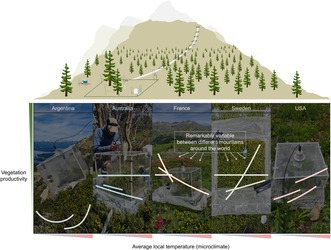

## 1 Introduction

Climate warming is currently reshaping how different components of the terrestrial carbon cycle function and interact (Bardgett et al. 2013; Naylor et al. 2020; Reich et al. 2020). Many of these changes may be gradual and linear. For example, plant biomass production in tundra ecosystems tends to increase linearly with increasing air temperature (Siregar et al. 2024; Van Der Wal and Stien 2014), at least until temperatures approach or exceed photosynthetic optima (Tieszen 1973). Yet not all responses follow such predictable trajectories. Some responses appear linear until a certain critical threshold is breached, whereupon the system transitions abruptly and non‐linearly to an alternative stable state (Kopp et al. 2016). In the last few decades, Earth system models have identified several system‐level changes, such as permafrost thaw, that could force systems beyond carbon thresholds and, in turn, accelerate climate warming (Wang et al. 2023).

Much of our understanding of vegetation thresholds and the land carbon sink derives from dynamic global vegetation models (DGVMs), which are a component of current Earth system models (Argles et al. 2022). At the global scale, DGVMs indicate that the land carbon sink has increased in the last 60 years, largely driven by CO_2_ fertilisation of tropical forests and warmer and longer growing seasons at high latitudes (Friedlingstein et al. 2025; Ruehr et al. 2023). Yet it is likely that increases in global plant carbon uptake will start to decelerate or reverse in coming decades as a consequence of increasing water, nutrient and temperature stress (Duffy et al. 2021; Liu et al. 2018; Wang et al. 2020). As these stresses change over time, along with associated shifts in insect herbivores and fire regimes, there is a strong possibility that ecosystems will approach thresholds in plant carbon uptake (Braghiere et al. 2023; Buermann et al. 2014). Predicting how these dynamics will change across different biomes is crucial to our understanding of global climate change and informing ecosystem management.

There is a long tradition in ecology of using elevational and latitudinal gradients to predict the responses of various biota and ecosystem processes to warming (e.g., Frenne et al. 2013; Hagedorn et al. 2019; Sundqvist et al. 2013). Although these space‐for‐time approaches have limitations (Fukami and Wardle 2005), such as not accounting for novel interactions or dispersal lags (Alexander et al. 2018), they nevertheless can provide a useful approximation of long‐term ecological change (Lovell et al. 2023). One complication is that these gradient studies rarely measure microclimate, which can be decoupled from the broader macroclimate change associated with elevation or latitude (Graae et al. 2012; Körner 2007). In our case, microclimate refers to the combination of local climate conditions in the 1–2 m^2^ surrounding a plant (Kemppinen et al. 2024). Effectively, the climatic conditions experienced by small organisms, such as ground‐layer plants and soil biota, vary markedly from macroclimatic conditions depending on, for example, their position in the landscape or their interactions with larger plants that can create a boundary layer of air that is partly decoupled from the more well‐mixed air above (Mazzotti et al. 2023; Zellweger et al. 2020). Thus, when studies find no relationship between ground‐layer carbon fluxes and macroclimate associated with elevation or latitude (e.g., Prager et al. 2021), it may not reflect a fundamental lack of any climatic control over these processes, but rather that the relevant climatic drivers have not been recorded at the appropriate spatial scale. A central goal of our study was to explore microclimate‐macroclimate decoupling as a potential driver of variability in plant carbon uptake.

Mountain ecosystems cover about 27% of the global land area (Nan et al. 2025) and have played a pivotal role in advancing climate change research, as they provide a wide array of climatic conditions across a relatively small area (Hagedorn et al. 2019; Tito et al. 2020). In particular, the transitions from forest to treeless alpine or subarctic vegetation—referred to as forest‐tundra gradients—are likely to be the site of substantial non‐linear changes in carbon cycling under future warming (Greenwood and Jump 2014; Mayor et al. 2017). A key area of research shows that large carbon stocks in subarctic tundra soils are vulnerable to increased decomposition under warming, driven not only by heightened microbial activity but also by shifts in microbial community composition favouring certain ectomycorrhizal fungi taxa (Clemmensen et al. 2021; Parker et al. 2015). Another key area focusses on range‐shifting plants, particularly the potential for expanding species, namely shrubs, to enhance both carbon uptake and losses (Mekonnen et al. 2021). Many studies have examined the drivers of tundra carbon fluxes, typically linking them to vegetation types and soil microclimate during measurement (e.g., Dagg and Lafleur 2011). However, few have explored these dynamics along environmental gradients (Virkkala et al. 2018). Among the limited studies using elevation gradients (Prager et al. 2021; Sloat et al. 2015; Wu et al. 2011), only one (Prager et al. 2021), which included 12 plots, had sufficient resolution to detect non‐linear patterns, yet it found no relationship between elevation and Net Ecosystem Exchange (NEE). The lack of empirical studies assessing for non‐linearity in the relationship between carbon fluxes and macroclimate represents an important knowledge gap, since thresholds in fluxes could have a major impact on the strength of the land carbon sink and its capacity to offset human carbon emissions (Friedlingstein et al. 2025). A second major knowledge gap is the absence of estimates for total ecosystem carbon uptake, including contributions from both trees and ground‐layer vegetation, along elevation gradients. Our study seeks to fill these gaps by examining how GPP varies across continuous forest‐tundra gradients and what this means for carbon cycling in a warming world.

There are potentially several drivers of non‐linearity in the climate responses of ecosystem GPP along forest‐tundra gradients. A non‐linear change in a response could be considered a ‘threshold’ if the change represents an abrupt state shift (Kopp et al. 2016). The most well‐studied state shift along forest‐tundra gradients is the treeline, that is, the low temperature limit for tree growth, often further limited by disturbance (Holtmeier and Broll 2018; Körner and Paulsen 2004). Treeline dynamics are poorly captured by current DGVMs—largely due to resolution and conceptualisation issues—and represent a considerable source of uncertainty in attempts to predict future carbon cycling (Nagy et al. 2023). Across treelines globally, Mayor et al. (2017) found a strong decline in ground‐layer leaf nitrogen (N) concentration with increasing elevation, indicating a possible decline in primary production. Even in the sparse subarctic forests of northern Sweden, trees contribute about half of the total leaf area in the ecosystem (Parker et al. 2020), so there is likely to be a sharp threshold of primary productivity at the treeline (Greenwood and Jump 2014). Recent work by Wang et al. (2025) suggests that the soil microbial biomass is also concentrated around the treeline, potentially reinforcing the disproportionate influence of forest‐tundra ecotones on carbon cycling in mountains. Other non‐linear mechanisms along forest‐tundra gradients could include geomorphic zones of unstable soil at high elevations (Eichel et al. 2023), frost‐heaving (Jonasson and Sköld 1983), and the range limits of insect herbivores (Parker et al. 2017) or dominant plant species with extreme functional contributions (Brun et al. 2022).

Our current understanding of forest‐tundra GPP is based on studies with limited spatial coverage, primarily from Alaska and Fennoscandia (Virkkala et al. 2018). As a result, we lack a comprehensive view of how plant carbon inputs change continuously along temperature gradients. This gap in knowledge contributes to substantial uncertainty when trying to generalise findings to ecosystems globally. At continental scales, ecosystems have evolved under diverse climatic and geological conditions, leading to considerable variation in ecosystem properties and the functioning of biota (McKenzie et al. 2024; Reeb et al. 2024). For example, conclusions about plant N cycling from European ecosystems may not apply to Australian ecosystems, where plants typically have low leaf N contents (Flores‐Moreno et al. 2023). These large‐scale uncertainties limit our ability to accurately assess ecological patterns and processes at the global scale. To address this gap, we established a series of forest‐tundra gradients across four continents to investigate how GPP may respond to future warming. Our sampling approach was designed to detect non‐linear ecosystem responses and the extent of coupling between macro‐ and microclimate. We measured leaf N per unit leaf area (N_area_) as a proxy for N‐rich photosynthetic machinery, and biomass as a proxy for leaf area, both of which are positively associated with photosynthetic capacity and could be valuable predictors of GPP (Wright et al. 2004). We tested two main hypotheses:

1. Whole‐ecosystem GPP is associated with macroclimate temperature (approximated by elevation) and increases non‐linearly with decreasing elevation;
2. Ground‐layer GPP is associated with microclimatic temperature and increases with warmer growing‐season temperatures.

We also explored the extent to which variation in ground‐layer GPP could be explained by leaf N_area_ and aboveground biomass. Overall, our study represents a globally coordinated effort to improve predictions of carbon cycling in mountain ecosystems. By using spatial gradients as proxies for temporal change, we gained insights into whether mountain regions worldwide are nearing non‐linear changes in their capacity for carbon uptake—findings with important implications for ecosystem services and the trajectory of global climate change.

## 2 Methods

### 2.1 Study Design

**FIGURE 1:**
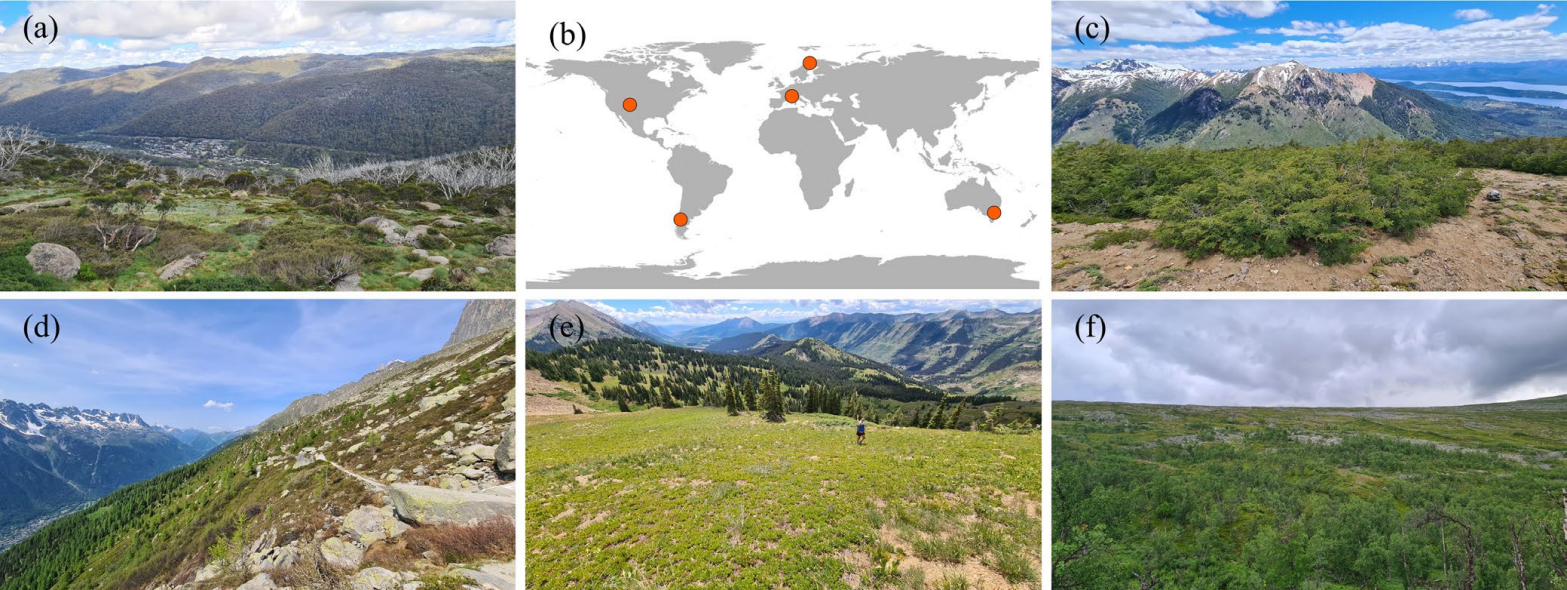
Global map of sampled regions (b), and example transect sites from (a) Australia, (c) Argentina, (d) France, (e) USA and (f) Sweden.

We conducted our study in mountainous regions of five countries: Australia, France, Sweden, USA, and Argentina (Figure 1). We selected mountain ranges that (1) were located outside the tropics to avoid complex cloud and precipitation effects (Halladay et al. 2012), and (2) supported subarctic or alpine tundra (treeless) vegetation at high elevations. None of the mountains that we selected have a region of permanent snow (nival zone) throughout the year.

In each country, we selected three replicate elevation gradients on independent mountains (> 4 km apart) and established transects of ten 10 × 10 m square plots along each gradient. Simulations indicate that using 10 levels along an environmental gradient is generally sufficient for detecting the true underlying response shape (Schweiger et al. 2016). Our transects crossed the treeline, with 3–5 plots established in subalpine/subarctic forests and 5–7 plots in treeless tundra vegetation (Table S1). For simplicity, we refer to vegetation above the treeline as “tundra” and vegetation below the treeline as “forest”. We visually determined the treeline along each gradient as the highest elevation limit of tree species (minimum 3 cm diameter at breast height), excluding small islands of trees or krummholz above this point that are subject to anomalous microclimatic conditions (Harsch and Bader 2011). The treeline varied in form among countries. For example, in Argentina, the “treeline” was the abrupt boundary formed by tall krummholz (Figure 1c), while in France, there were often isolated seedlings and saplings above the main forest treeline (Figure 1d). The various constraints of treelines (see Hansson et al. 2023 for a review) were not an explicit focus of our study, since we aimed to predict response trajectories of carbon uptake along gradients rather than rates of tree expansion or responses at specific times in the future. Our space‐for‐time design assumes that tree species were approximately in equilibrium with their temperature niche, and if this assumption is violated, then future responses cannot be assumed to mirror present‐day spatial patterns.

Within each transect, plots were evenly spaced by elevation across the full transect, but adjusted if the area was highly disturbed (e.g., by digging animals), anomalously moist (e.g., a snowmelt stream), or had a high cover of exposed rock. Consequently, fewer forest plots were established when forest covered a smaller proportion of the full elevational gradient (Table S1). For the most part, we did not avoid transitions in the vegetation community, as these can be driven by the temperature gradient (Sundqvist et al. 2013). The one exception was northern Sweden, where we maintained a heath community across all transects where possible (see Section 2.2). It was not always possible to maintain the same cardinal aspect for an entire transect, and so we allowed variation of up to 135° (i.e., 67.5° in either direction from the average aspect of the transect).

For detailed information about site characteristics, including climate, vegetation, and herbivores, see Appendix S1.

### 2.2 Plant Survey and Traits

We sampled during the peak growing season of each country (Table S1), that is, the point where most plants had reached maximal leaf expansion but not yet started to senesce. In each plot, we surveyed plant species composition using a grid of 50 point‐intercepts spaced evenly across the entire area. Whenever a plant touched a cylindrical baton (8 mm diameter) at a sampling point, we recorded a presence of that species. We then derived the cover of plant species as the proportion of presence ‘hits’ out of the total 50 points. Overhead tree species were recorded as a presence when the sampling point fell within the canopy extent.

From the plot survey, we measured plant functional traits for all species that exceeded 10 ‘hits’ (i.e., 20% cover, allowing for vertical overlap of species). This arbitrary cut‐off is based on the notion that species with higher abundances have a greater influence on ecosystem functioning (Grime 1998). In a typical plot, there were 2–6 dominant ground‐layer plant species (> 10 hits). However, if there was only one dominant ground‐layer species above the cut‐off, we also measured the species with the next highest number of hits.

We measured two traits to derive leaf N_area_: specific leaf area (SLA) and leaf N concentration. We first collected unblemished leaf samples from at least 10 individuals per dominant species within a plot. Area was calculated in ImageJ (Schneider et al. 2012) using flattened scans of fresh leaves between two acrylic sheets. Leaves that were rolled or keeled were unravelled prior to scanning and cut into pieces if there was any overlap in leaf area. Since our focus was on carbon cycling rather than the hydraulic functioning of the leaf, we removed leaf petioles before scanning (Pérez‐Harguindeguy et al. 2013). The area of conifer needles was measured as the two‐dimensional projected area, which aligns with measurements of Leaf Area Index (LAI) and leaf‐level fluxes. We refer to projected area as the one‐sided area projected onto a plane that is perpendicular to the incoming light direction. For non‐vascular plants and lichens, we measured the flattened projected area of the whole intact organism, which can be functionally equivalent to vascular plant leaves because non‐vascular plants and lichens are almost entirely a photosynthetic surface (Mallen‐Cooper and Cornwell 2021). In these scans of non‐vascular plants, we separated broader fronds and stems but allowed for some overlap of minute leaf tissues. Once area was calculated, the leaves were dried in an oven at 65°C for 48 h and then weighed. SLA, or its non‐vascular equivalent, was derived from the ratio of area to mass. Finally, a sample of leaves from at least 10 individuals was dried in an oven at 65°C for 48 h, crushed in a ball mill, and analysed for N concentration using an Elemental Analyzer (Flash EA 2000, Thermo Fisher Scientific, Bremen, Germany) and Isotope Ratio Mass Spectrometer (DeltaV, Thermo Fisher Scientific, Bremen, Germany). Leaf N per area (N_area_) was calculated as leaf N content divided by SLA. For ground‐layer vegetation, community‐weighted trait means were calculated at the plot level using species abundances (point‐intercept hits) from the survey.

In each plot, we also measured the diameter at breast height (DBH) of all trees (here defined as those with DBH > 3 cm), avoiding any anomalous protrusions such as burls. In multi‐stemmed trees (e.g., *Eucalyptus pauciflora* ), we summed the squared DBHs of each stem that reached breast height (1.3 m) and derived the square root of this sum. In stems with a prostrate component, we measured the DBH at 1.3 m from the tree base.

We measured aboveground plant biomass of the ground‐layer vegetation in four random 18 × 18 cm squares within each plot. Biomass was cut at ground level and along the square border, and then dried in an oven at 65°C for 48 h before weighing. Lichens and bryophytes were included in the sample, as was necromass that remained attached to living biomass.

### 2.3 Ground‐Layer GPP Measurements

To measure the ground‐layer carbon flux, we used a clear acrylic chamber (40 × 40 × 40 cm) with a removable lid for ventilation (Figure S1). The chamber was sealed at its base with a polypropylene plastic skirt, metal chain, and often rocks (Grau‐Andrés et al. 2021). Inside the chamber, we placed a Photosynthetically Active Radiation (PAR) sensor (Apogee Instruments, USA) elevated above the vegetation and oriented vertically. We also used two computer fans (80 mm diameter) mounted internally on opposite sides of the chamber to circulate air during measurement.

In each plot, we measured ground‐layer carbon fluxes in three random locations, allowing for random repositioning if the surface was very uneven. In those locations, we recorded headspace CO_2_ concentrations inside the chamber every 5 s for 2‐min periods using a GMP343 CO_2_ probe (Vaisala, Finland; Grau‐Andrés et al. 2021; Sørensen et al. 2019). At a single location, we conducted four measurement periods at different shading levels, so as to construct a light response curve. Shading levels ranged from complete darkness, capturing only ecosystem respiration (ER), to maximum available sunlight. Dark and intermediate shading levels were created using plastic or other material coverings that varied in opacity. Between each measurement period, we unfastened the lid and allowed the chamber to ventilate for 1 min.

The rate of change of [CO_2_] over the 2‐min period was derived from the slope of a robust linear regression, which down‐weights outlying values that may arise from heterogeneous bursts of CO_2_ emission from the soil. Some measurement periods were manually trimmed if we recorded issues with sealing or changes in insolation. The rate of change of CO_2_ was converted to μmol m^−2^ s^−1^ using the ideal gas law, temperature recorded by the CO_2_ probe, and air pressure based on altitude (Street et al. 2007).

At each location, comprising four shading levels, we modelled the relationship between the CO_2_ flux and PAR (i.e., the light response curve). Using the *nlme* (version 3.1‐163; Pinheiro and Bates 2025) and *photosynthesis* (version 2.1.4; Stinziano et al. 2021) R packages, we fit linear, exponential and rectangular hyperbola models, and selected the best‐fitting model according to the Akaike information criterion (AIC).

Rather than normalising all fluxes to a single PAR value (e.g., 600 μmol m^−2^ s^−1^; Sundqvist et al. 2020), we adopted a novel approach to account for light interception by tree canopies in forest plots. Vegetation underneath a canopy is typically exposed to a much lower light intensity than vegetation in an open tundra environment (Sercu et al. 2017). To account for the lower light availability in plots with trees, we derived a plot‐level estimate of canopy light transmittance. This estimate involved selecting at least 10 isolated (i.e., with no adjacent trees) individuals of each tree species, and measuring PAR at 20 random points underneath an individual's canopy (PAR_canopy_), whilst simultaneously measuring PAR in open full‐sun conditions (PAR_open_), during the middle of the day (10:00–14:00). For each individual tree, we calculated the mean proportional light transmittance (proportion of light remaining after canopy interception, PAR_canopy_/PAR_open_) based on the 20 paired replicates. We then computed a generalised additive model (GAM) of the relationship between canopy light transmittance and diameter at breast height (DBH) as measured in the same trees. Finally, we derived a plot‐level estimate of canopy light transmittance based on the mean DBH of all trees in the plot, and, if multiple tree species were present, the relative abundance of each species. We then normalised all plots to 600 PAR in open‐sky conditions, which, in forest plots, was reduced to a “realised PAR” of 600 multiplied by the canopy transmittance coefficient, which ranged from 0.16 to 0.95. While all PAR measurements were made during the middle of the day and typically close to the middle of summer, some additional error was likely introduced due to seasonal, diurnal and latitudinal variation in the solar zenith angle.

Ground‐layer GPP was calculated by subtracting the respiration (full shade) measurement from the net flux (net ecosystem exchange) at 600 PAR or the realised equivalent in forest plots. Note that in Argentina, ground‐layer fluxes were only measured across two transects (Catedral and Lopez).

### 2.4 Tree GPP Upscaling

From 10 to 20 trees of each species that were nearby but not within plots, we collected branches (approx. 60 cm long) and immediately re‐cut them under water to minimise cavitation. Branches were collected from a range of crown heights and light exposures, and contained leaves from a variety of age classes, so that canopy‐level estimates would be representative of entire trees under natural field conditions. In this way, our goals differ from physiological studies, which typically aim to quantify the maximum potential photosynthetic capacity (e.g., Azuma et al. 2024).

We used a leaf chamber device (Li‐6800; LICOR Biosciences, USA) to produce light response curves for individual leaves, or a group of needles, from different tree species. One random leaf, or a section of needles, from each branch was measured in the leaf chamber within 15 min of collection. For each response curve, a leaf was exposed to a series of decreasing photosynthetic photon flux densities (PPFD), namely 1200, 800, 600, 400, 200, 100 and 0 μmol m^−2^ s^−1^, using the inbuilt blue‐red LED lamp. At each PPFD, CO_2_ fluxes were allowed to stabilise for a maximum of 2 min before a measurement was logged. Flow rate was set to 500 mL min^−1^, and reference CO_2_ concentration was set to 420 μmol mol^−1^. Chamber temperature and relative humidity remained at ambient levels, but were monitored to ensure that they remained within a reasonable range. All broadleaved species covered the entire area of a 3 × 1 cm chamber. However, for needle‐leaved trees, we placed a group of 4–8 non‐overlapping needles inside a 3 × 2 cm chamber, and after measurement, trimmed the needles so that only the part inside the chamber remained (Bryant et al. 2022). We then calculated the two‐dimensional projected area of this measured part using ImageJ, and corrected the flux accordingly. We used the projected, rather than three‐dimensional, area for needles because it corresponds to the way that LAI is estimated. Note that in Argentina, we were unable to use the leaf chamber device, and instead used published light response curves from Martínez Pastur et al. (2007), excluding the low light intensity treatment as it would not be representative of canopy trees.

Light response curves were fitted to these leaf chamber measurements using the same methods as ground‐layer fluxes, taking the best‐fitting option from linear, exponential, and rectangular hyperbola models. Final leaf estimates of respiration and GPP were extracted from these curves at 0 and 600 PAR respectively, in order to match ground‐layer calculations.

The LAI was estimated in at least 10 isolated trees of each species using an optical device (LAI‐2200, LICOR Biosciences, USA), which automatically computes LAI—or, more precisely, Plant Area Index—based on light interception at five different zenith angles. These ‘calibration trees’ were located within 1 km of a transect, and were not contained within plots. We collected 10 repeated measurements underneath the canopy of each tree, and restricted the field‐of‐view so that it captured only the tree canopy and no surrounding sky. There are two main limitations to this method: (1) it inadvertently captures woody area, resulting in an overestimate of LAI, and (2) it does not account for foliage clumping, resulting in an underestimate of LAI (Zhu et al. 2018). In mixed forests in south‐eastern Germany, Zhu et al. (2018) found that the combined error from woody area and clumping generally falls in the range of ±25%, with broadleaf trees typically showing dominant woody effects (overestimated LAI), and conifers showing dominant clumping effects (underestimated LAI). Additional error may have been introduced by variable leaf inclination angles, with more vertical leaves leading to an underestimated LAI and more horizontal leaves leading to an overestimation (Li et al. 2024).

To estimate the GPP of trees in an individual forest plot, we multiplied the mean species leaf GPP (μmol m^−2^ s^−1^) from leaf chamber measurements by the horizontal (% tree cover) and vertical (LAI, m^2^ m^−2^) extents of trees within the plot (Equation 1).

(1) $$\mathrm{GP}P_{\text{trees}}=\mathrm{GP}P_{\text{leaf}}\times \text{cover}\times \mathrm{LAI}$$

Tree cover was the abundance recorded in the plant survey (see Section 2.2). LAI was adjusted to plot‐level DBH, by first fitting a generalised additive model (GAM; thin plate regression spline with a maximum of five basis functions; see Section 2.6) of the relationship between LAI and DBH in all calibration trees, which outperformed a linear model according to AIC. We then used this model to scale LAI by the average DBH in each plot, although it should be noted that LAI‐DBH relationships were generally weak for most tree species (Figure S2). Finally, if there were multiple tree species in a plot, we derived an average leaf GPP that was weighted by species relative abundance from the plant survey. We also conducted an error propagation analysis to assess the full extent of variability in tree GPP estimates. For this analysis, we simulated the accumulated variation in LAI‐DBH relationships and leaf‐level fluxes using non‐parametric resampling (Figure S3). That is, for each tree species in each plot, we took a random draw of the replicate leaf flux values, and added a random amount of noise to the LAI‐DBH calculation based on the residual standard deviation in the LAI‐DBH model. We then estimated tree GPP using these simulated values, and repeated the process 50 times to produce a simulated error distribution.

It is worth noting that respiration by tree roots is captured by the ground‐layer flux measurements, which could contribute to reduced estimates of ground‐layer GPP in forest plots. For clarity, total ecosystem GPP was estimated according to Equation (2), with R_soil_ including tree root respiration, and no term capturing stem or branch fluxes.

(2) $$\begin{array}{c} \mathrm{GP}P_{\text{ecosystem}}=\mathrm{GP}P_{\text{trees}}+\mathrm{NP}P_{\text{ground}-\text{layer}}-R_{\text{soil}} \end{array}$$

### 2.5 Microclimate

A microclimate sensor (TOMST TMS‐4) was placed in a central location in every plot. The sensor recorded soil moisture in the upper 14 cm and temperature at three levels (soil surface, 8 cm below, and 15 cm above) every 15 min (Wild et al. 2019). In Argentina, all sensors in the tundra zone were placed under a few rocks to protect from potential disturbance, which may have created a buffered microclimate around the sensor. In all forest plots, we also attached a second sensor (TOMST Thermologger) to a tree on the equator‐facing side of the trunk and at a height of 2 m. Both types of sensors had shields to protect from direct sunlight, although the upper shield of the ground‐based sensors was sometimes removed by snow movement or animal disturbance, so we chose to exclude the uppermost temperature data from these sensors.

From the sensor data, which was downloaded after about 1 year, we computed the following daily temperature statistics: maximum and minimum temperature, mean temperature (mean of maximum and minimum), temperature range (difference of maximum and minimum), and mean soil moisture. The values of moisture provided by the sensor were transformed to gravimetric soil moisture by calibrating samples of local soils from the tundra and forest of each country. For the calibration, we used unsieved soils collected from at least one tundra and forest plot per country, with a greater number of calibration samples taken in regions with highly heterogeneous soil types. We then created a gradient of moisture by sequentially adding water to the same sample and gently stirring until moisture was homogenous. At each moisture level, we recorded moisture using both the sensor and conventional oven drying methods (drying oven at 105°C until constant mass), and then derived the best linear or exponential model fit based on the AIC to transform recorded values to gravimetric moisture. We also estimated the start and end of the growing season using the reduced temperature range underneath snow cover as a proxy. We defined the dormant season as a period of < 0.3°C daily temperature range at the soil surface that lasted longer than five consecutive days (Figure S4).

We used a range of imputation techniques to fill in missing data resulting from broken sensors or too few days of recording. If data were not available for one full growing season, we used cyclic generalised additive mixed models (GAMMs; Wood 2011) with a temporal autocorrelation term to impute daily temperature statistics for the missing days (maximum 90 days). For broken sensors, we used the closest available information to impute the missing data. For example, in forest plots, we imputed temperatures using the tree sensor in the same plot and the relationship between air and soil temperatures in a nearby plot (fitted with a generalised additive model; *n* = 2 plots). In one plot, we were able to use data from previous years and its relationship to a nearby sensor. The final option we used was to average the sensor data from the two closest plots (*n* = 7 plots).

Next, we calculated three microclimate statistics for the entire growing season period: mean temperature, growing degree days (the annual number of days in which the maximum temperature exceeded 5°C), and mean soil moisture. The number of growing degree days relates to the approximate low temperature limit of plant photosynthesis and bud opening (Grace et al. 2002), and was not correlated with mean temperature (Spearman *ρ* = 0.02).

To explore the extent of cold air pooling in each transect as a potential driver of microclimate decoupling (Pastore et al. 2024), we selected the highest and lowest plot in the forest. The reason for only including forest data was that temperature comparisons across the entire forest‐tundra gradient could be confounded by the buffering effects of trees. We then derived the percentage of temperature inversions from tree Thermologgers (15 min timesteps; Joly and Richard 2022), that is, the relative number of timesteps when temperature was higher in the higher elevation plot.

### 2.6 Data Analysis

We considered each transect to be an independent response curve derived from a series of non‐independent levels (plots). For this reason, we chose to fit generalised additive models by transect, rather than other non‐linear approaches that rely on many independent replicates along a single environmental gradient, such as change point models (Ficetola and Denoël 2009).

All modelling was conducted using the *mgcv* R package (version 1.9‐1; Wood et al. 2016), and statistical assumptions and fit were assessed using the *gratia* package (version 0.9.2; Simpson 2024). When GPP was the response variable, we used air temperature and soil moisture during measurement as control variables in the model. To display results in two dimensions, we predicted ground‐layer GPP for a standard gravimetric soil moisture of 50% and a standard air temperature of 18°C, which represent, respectively, the global median of moisture and maximum daily temperature during the growing season as detected by our microclimate sensors (see Figures S5 and S6 for examples of different standard temperatures). Standardising conditions in this way allows for comparisons among continents, but comes with the caveat that the predicted GPP might not represent the realised GPP under local conditions. Note that pseudoreplicates within plots were averaged prior to modelling.

GAMs were fit with a thin plate regression spline, with a null space penalty that effectively shrinks weak relationships (high smoothing parameter *λ*) to a horizontal line. We also restricted the main smooth to five basis functions (and control variables to two) to limit overfitting. As such, we opted for a highly conservative approach to avoid Type 1 errors. Finally, we used a Tweedie error distribution with a log link function, as this was the best fit for all models.

We applied the inbuilt Wald‐type test within *mgcv* to indicate whether fitted curves were significantly different from a horizontal line (Wood 2013). The *p*‐values arising from these tests should be considered an approximate indicator. We also used the effective degrees of freedom (EDF) of fitted smooths as an indicator of non‐linearity. Following previous work, we interpret an EDF close to 1 as linear, and an EDF > 2 as strong evidence of non‐linearity (Bringmann et al. 2017; Hunsicker et al. 2016; Shadish et al. 2014). While GAMs cannot specifically identify a ‘threshold’ as in a change point model, we chose to interpret strongly non‐linear shifts in carbon uptake as thresholds if there was a plausible consistent shift in the vegetation community (e.g., treeless tundra to forest).

## 3 Results

**FIGURE 2:**
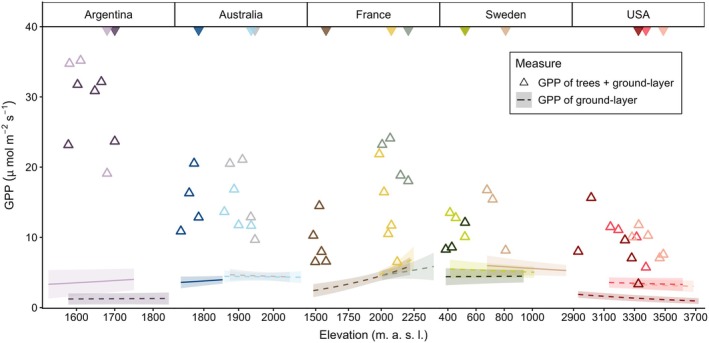
Modelled relationships (±95% CI) between ground‐layer vegetation Gross Primary Productivity (GPP, based on flux chamber measurements and standardised to 600 PAR open‐sky conditions) and elevation across forest‐tundra gradients. Open triangles represent point estimates of total GPP, that is, when tree carbon uptake is added to the ground‐layer GPP based on up‐scaled leaf fluxes (see Section 2). Treelines are indicated by solid triangles at the top of each panel, significant relationships (at *p* < 0.05; see Table S6 for statistical results) are shown as a solid line, non‐significant relationships are shown as dashed lines, and different colours represent different transects (see Figure 3 for colour codes).

Unsurprisingly, as the ecosystem transitions from tundra to forest at the treeline, our estimates of total ecosystem GPP indicate a sharp non‐linear increase in productivity for virtually all transects globally (Figure 2). However, among countries, there were sizeable differences in the magnitude of GPP responses to macroclimate, suggesting strong context dependence at the regional scale. The strongest increases in GPP at the treeline were estimated in the *Nothofagus pumilio* forests of Argentina, the *Eucalyptus pauciflora* forests of Australia and two transects in France that were largely dominated by *Larix decidua* (Aiguille and Nevache). These three tree species were all characterised by high leaf GPP (mean > 10 μmol m^−2^ s^−1^) and relatively low LAI (< 2.5; Table S2). By contrast, other trees such as *Picea engelmannii* and *Abies lasiocarpa* (dominant in the subalpine zone of our transects in the USA) supported high amounts of foliage (LAI > 2.5) but substantially lower leaf GPP (mean < 5 μmol m^−2^ s^−1^).

Subalpine forests in both the USA and Argentina supported relatively sparser ground‐layer vegetation (mean 73% and 39% cover, respectively) compared to other countries (> 95%; Table S3). It is also worth noting that the needle‐leaved trees in the USA intercepted a particularly high amount of light (> 80% interception of PAR in trees with the mean DBH of the species; Table S2), while mountain birch in Sweden produced far thinner canopies (54% interception at mean species DBH; Table S2). In terms of the horizontal structure of trees, forests in Argentina were characterised by relatively closed canopies (mean 86% canopy cover), while the most open forest canopies were observed in Sweden (mean 46% canopy cover).

**FIGURE 3:**
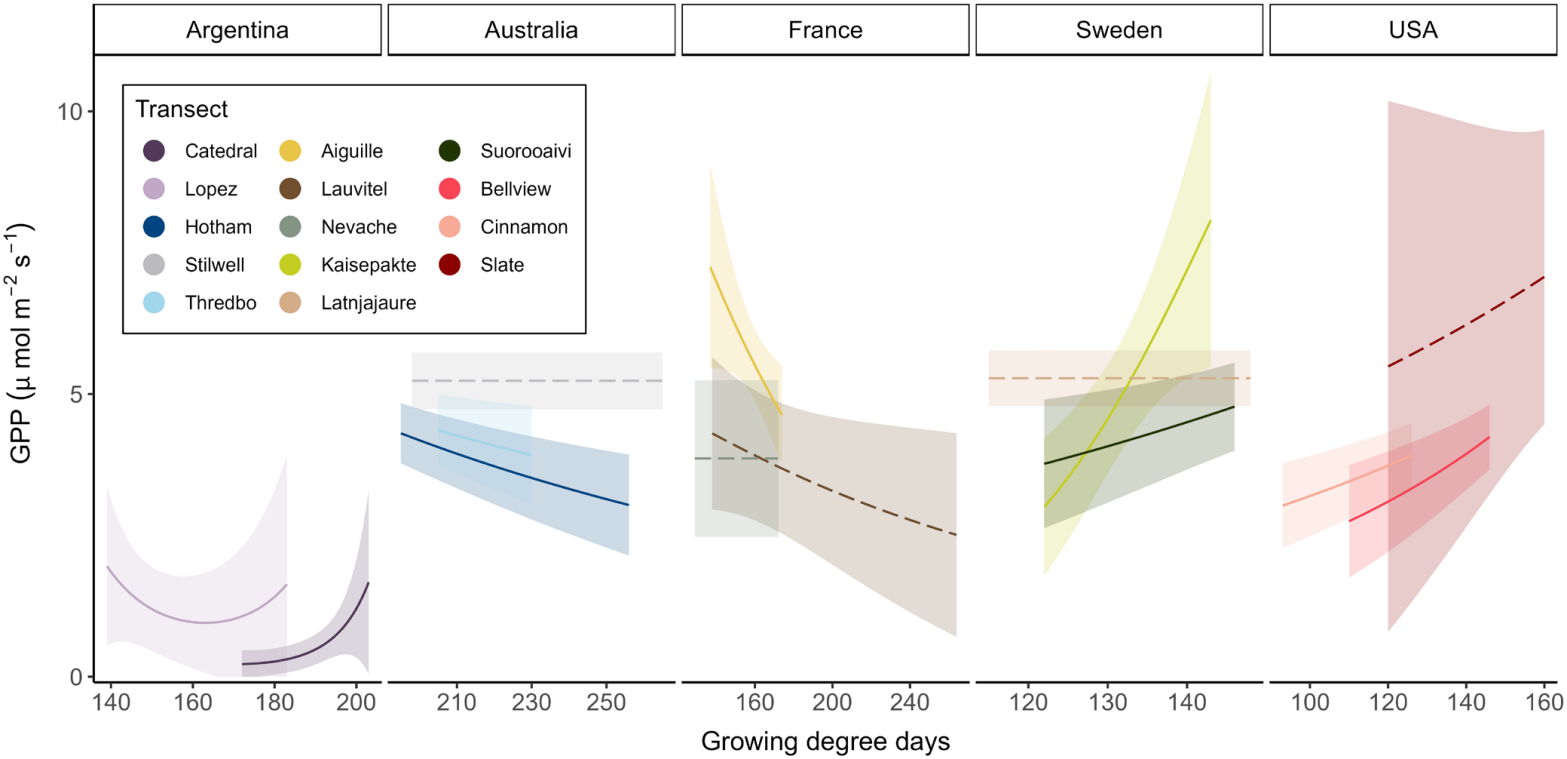
Modelled relationships (±95% CI) between ground‐layer vegetation Gross Primary Productivity (GPP, standardised to 600 PAR open‐sky conditions) and the annual number of growing degree days (> 5°C maximum temperature at ground level) across forest‐tundra gradients. Significant relationships (at *p* < 0.05; see Table S7 for statistical results) are shown as a solid line, non‐significant relationships are shown as dashed lines, and different colours represent different transects. Raw plot‐level data is not shown since it does not reflect the standard conditions used to compare GPP among transects (18°C air temperature and 50% soil moisture; see Section 2).

Despite finding limited evidence that ground‐layer GPP varied across elevation (Figure 2, Table S4), there was some evidence that it frequently varied with microclimate, particularly the number of growing degree days (Figure 3, Figure S7, Tables S5–S7). In some countries (Sweden, USA), GPP was typically higher in microclimates with more days available to photosynthesise, while the opposite pattern was observed along some transects in France and Australia. In several transects, we observed a significant relationship between ground‐layer GPP and mean soil moisture (Figure S8), with a wide array of response shapes indicating, again, strong context dependence at both local and continental scales. Notably, virtually all ground‐layer GPP relationships with microclimate were linear or close to linear (EDF < 2; Tables S5–S7), with the exception of some transects in Argentina.

**FIGURE 4:**
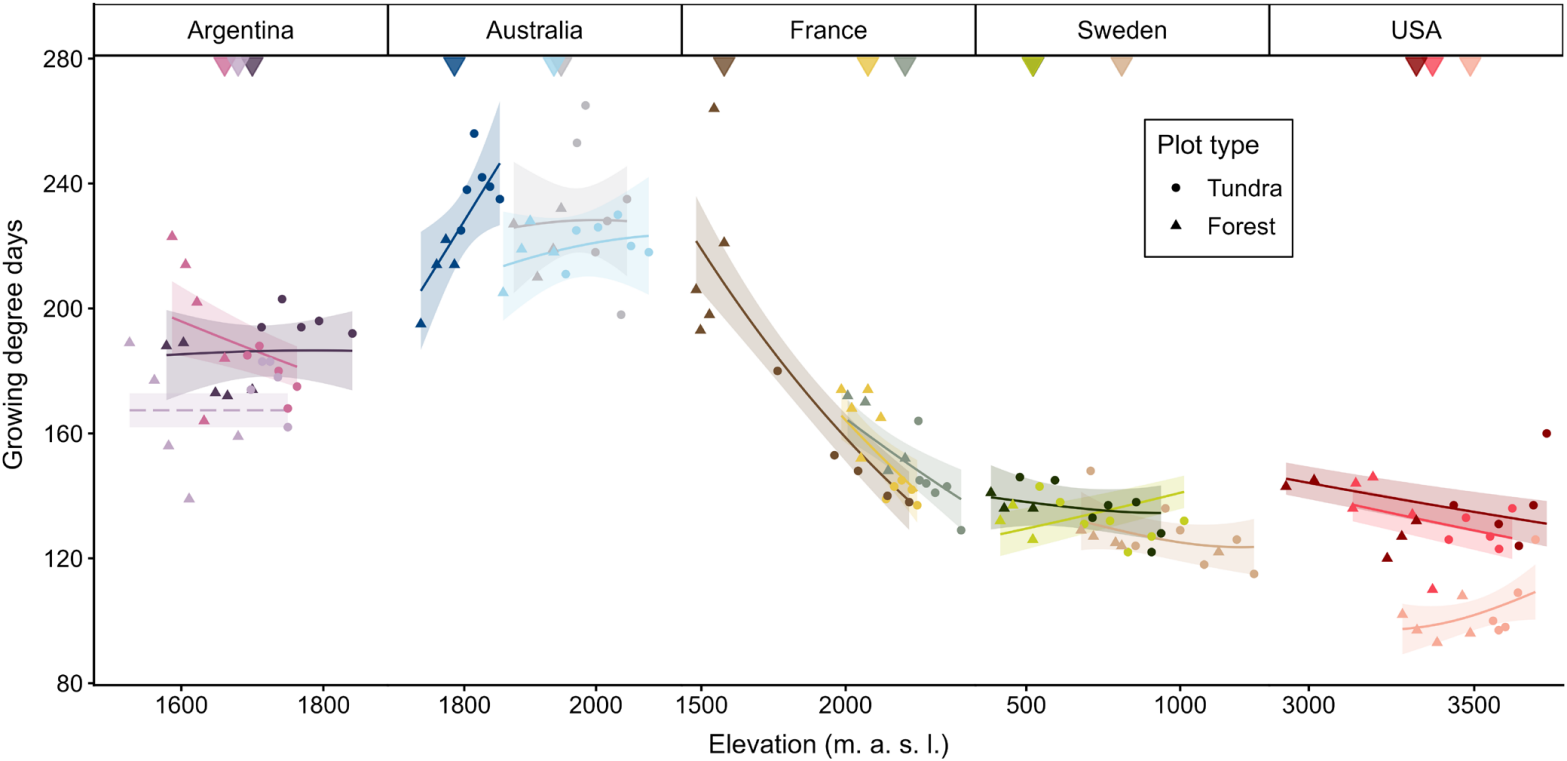
Modelled relationships (±95% CI) between the annual number of growing degree days and elevation across forest‐tundra gradients, with raw plot‐level data shown as points. Treelines are indicated by solid triangles at the top of each panel; significant relationships (at *p* < 0.05; see Table S2 for statistical results) are shown as a solid line, non‐significant relationships are shown as dashed lines, and different colours represent different transects (see Figure 3 for colour codes).

The relationships between microclimate temperature and elevation varied widely among transects, even within the same country (Figure 4, Figure S9, Table S8). For several transects, such as those in France and the USA, the annual number of growing degree days decreased with increasing elevation, as expected (Figure 4). Conversely, several transects showed a neutral or opposite pattern, with more growing days at higher elevations (Figure 4). Relationships between elevation and the mean growing season microclimate temperature were similarly divergent both within and among countries (Figure S9). Only one transect (Hotham, Australia) showed a clear pattern of longer snow pack duration with decreasing elevation (Figure S10). When assessing for cold air pooling, we found that temperature inversions occurred > 10% of the time for most transects, and > 40% of the time in some transects in Sweden and the USA (Table S9).

**FIGURE 5:**
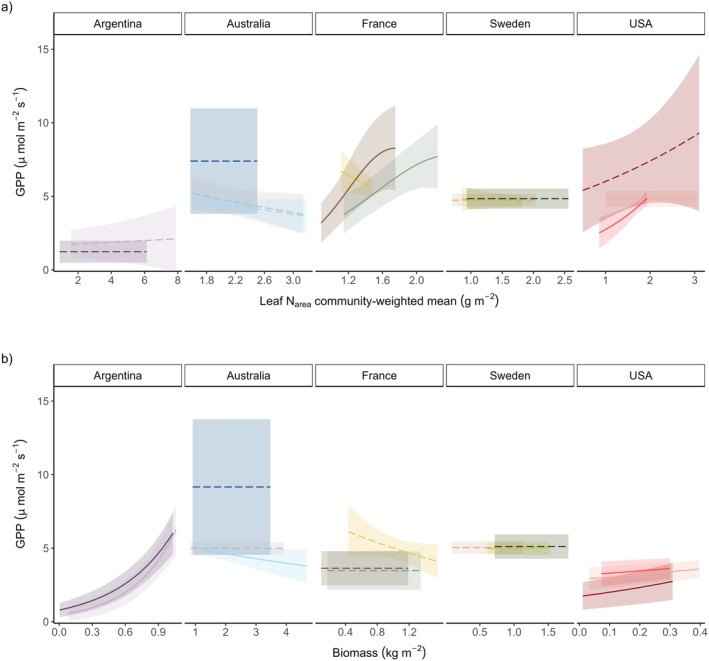
Modelled relationships (±95% CI) between Gross Primary Productivity (GPP, standardised to 600 PAR open‐sky conditions) and (a) leaf nitrogen per area (N_area_), and (b) aboveground biomass in ground‐layer vegetation (across all forest and tundra plots). Significant relationships (at *p* < 0.05; see Tables S10 and S11 for statistical results) are shown as a solid line, non‐significant relationships are shown as dashed lines, and different colours represent different transects. Raw plot‐level data is not shown since it does not reflect the standard conditions used to compare GPP among transects (18°C air temperature and 50% soil moisture; see Section 2).

Associations between ground‐layer GPP and both leaf N_area_ and biomass were limited and revealed strong context dependence (Tables S10 and S11). For example, GPP was positively associated with leaf N_area_ in two of the transects in France, but negatively associated in one (Figure 5a). Similarly, biomass played a role in moderating GPP in Argentina and the USA, where greater biomass was associated with higher GPP, while one transect in Australia showed the opposite pattern (Figure 5b).

## 4 Discussion

Using a global network of forest‐tundra gradients, we found that relationships between carbon uptake and elevation were remarkably inconsistent both among and within different mountain regions of the world. The one global uniformity was the outsized contribution of trees to GPP, suggesting that macroclimate‐driven tree expansion, if it is realised, could drive a sharp increase in carbon uptake in these ecosystems in the future. In about half of the sampled gradients, the inconsistency in ground‐layer GPP was associated with a decoupling of fine‐scale microclimatic variation from the broader macroclimate change across elevation. We also found evidence that leaf N and biomass sometimes moderated ground‐layer GPP in these gradients. Overall, our findings indicate that future warming is likely to affect carbon cycling on different spatial scales, such that trees will respond to macroclimate change while ground‐layer plants are more likely to respond to microclimate change. In some cases where trees drive macro‐ and microclimate decoupling, it could be that ground‐layer plants are buffered from broader macroclimate warming (Zellweger et al. 2020), although they may be potentially vulnerable to abrupt events that compromise the canopy such as insect outbreaks or drought (De Frenne et al. 2021; Parker et al. 2017).

Our results indicate that the warming‐induced shift from tundra to forest vegetation represents a critical threshold, beyond which ecosystem C uptake abruptly increases. In most countries, we found that tree cover led to two‐ to three‐fold increases in whole‐ecosystem GPP, relative to nearby tundra plots (Figure 2). This finding reinforces the obvious point that trees can support substantially greater leaf area per unit ground area than ground‐layer plants (Parker et al. 2020), and have an outsized impact on whole‐ecosystem C uptake. Even so, in ecosystems where treeline expansion is accompanied by altered rhizosphere processes, the enhanced decomposition of organic matter may still lead to a net loss of carbon overall (Jonsson et al. 2025; Parker et al. 2021). However, depending on other constraints such as dispersal and herbivory, the extent of some mountain tree species may lag behind their temperature niche as it expands upslope (Hansson et al. 2023; Lu et al. 2021). For example, *Eucalyptus pauciflora* treelines in mainland Australia are lagging far behind expected range shifts, likely due to poor seed dispersal and an increase in fire frequency (Fairman et al. 2017; Green and Venn 2012; Naccarella et al. 2020). In these Southern Hemisphere ecosystems, it may be many decades before tree‐driven increases in ecosystem carbon uptake are realised, if at all. Importantly, forest carbon uptake is shaped by both forest structure, such as tree density, and species‐level traits, such as leaf flux rates, total leaf area and leaf N (Reich 2012; Schall et al. 2018). We found substantial variation in both these factors among transects and regions, indicating that increases in primary productivity with tree expansion are likely to vary widely among canopy species.

There is growing recognition that small biota, such as herbaceous plants, tend to be controlled more strongly by microclimate than macroclimate (Haesen et al. 2023; Körner 2023; Lembrechts et al. 2018). Generally supporting this assertion, we found that ground‐layer plant carbon uptake responded more frequently to microclimate than to elevation. On a biophysical level, it is ultimately light at leaf level and fine‐scale temperatures surrounding leaf, stem, and root tissue that control photosynthetic efficiency and respiration in plants, given adequate water and nutrient availability (Wang et al. 2017). Fine‐scale temperatures can be modified by local topography, neighbouring plants, and an individual plant's own adaptations such as a compact growth form (De Frenne et al. 2021; Opedal et al. 2015; Sklenář et al. 2016). In tundra‐forest ecosystems, the viability of an individual's carbon budget is largely determined by the duration of time that leaves are photosynthetically active (Körner 2023; Wieser et al. 2005). In most of our transects in Sweden and the USA, a longer growing season was associated with higher instantaneous rates of GPP in ground‐layer vegetation, possibly because species in plots with a greater number of growing degree days have larger annual carbon budgets and can therefore invest in greater photosynthetic assets such as leaf area and photosynthetic enzymes. Additionally, a lower number of growing days is likely to correlate with greater frost stress and increased carbon allocation to below‐ground stems (Doležal et al. 2024; Makoto and Kudo 2020), potentially diverting carbon away from leaf area and photosynthetic machinery. However, we observed the opposite pattern in some Australian transects, and those plots with a greater number of growing days tended to be in the alpine tundra. This result suggests that despite a greater duration of growing time above the treeline, there may be other constraints on tundra plants in this region, such as low nutrients or mechanical stress by wind that limit carbon allocation to photosynthetic tissues (Doležal et al. 2021; Treby et al. 2024).

Our results indicate that the variability in ground‐layer carbon responses could be partly attributed to microclimates that were decoupled from the broader macroclimate gradient associated with elevation. This decoupling can be driven by several microclimatic phenomena. The first is a microclimate effect induced by trees, through shading and the creation of a buffered boundary layer of air that is, at least partially, protected from wind convection (Zellweger et al. 2020). These effects can, in turn, increase snowpack accumulation and duration (Mazzotti et al. 2023; Renard et al. 2016), although this was only evident in one of our gradients that had a poleward aspect (Hotham, Australia). The strong decoupling of elevation and microclimate in the USA could partly be explained by the dense shading canopies of *Abies lasiocarpa* and *Picea engelmannii* . Aside from canopy trees, it is equally possible that tundra plants, particularly dwarf shrubs, generated a buffered microclimate at ground level (Körner 2021). We also found evidence that cold air pooling is likely to be driving some degree of microclimate decoupling in most gradients, particularly in the USA, which aligns with recent reports of frequent cold air pooling in North American mountain systems (Pastore et al. 2024). Indeed, one of our sites in Australia (Stilwell) is known to have an inverted treeline due to cold air pooling (McDougall and Walsh 2007), although our gradient did not include the treeless valley at this site. Finally, topographic effects may have influenced microclimatic conditions in our plots, although this is less likely since the transects were arranged to minimise variation due to aspect and topographic anomalies.

There are four main mechanisms that could drive GPP variation along temperature gradients: (1) direct temperature effects on the rate and duration of photosynthetic activity over time, (2) species turnover, (3) intraspecific trait variation, and (4) external processes that impact leaf area (e.g., herbivores, frost damage). We used the community‐weighted leaf N_area_ trait to collapse turnover and intraspecific variation into a single dimension based on theoretical foundations that stipulate that greater amounts of N‐rich photosynthesis enzymes will increase a plant's capacity to fix C (Kattge et al. 2009; Reich et al. 1998). Yet few transects showed a relationship between leaf N_area_ and GPP (Figure 5a). This may reflect N‐rich compounds and metabolites other than those involved in photosynthesis having a larger‐than‐expected contribution to leaf N (Cooney et al. 2021; Raven 2013). For example, the amino acid proline is particularly important in forest‐tundra ecosystems, conferring freezing resistance in plant cells (Fedotova and Dmitrieva 2016; Ma et al. 2015). Similarly, we targeted leaf area as a moderator, using biomass as a proxy, and this dimension captures species turnover, intraspecific variation, and other processes that affect leaf area such as herbivory (Moreira et al. 2018). We found evidence that plots with greater ground‐layer biomass supported higher rates of GPP, but only in regions where biomass was relatively low, that is, Argentina and the USA (Figure 5b). The strong relationships in Argentina demonstrate the diversity of vegetation types in this region, ranging from virtually no ground‐layer plant cover in the krummholz zone (where the dominant tree species grows in a dense shrub‐like form at the treeline) to productive low shrubs such as *Maytenus disticha* in the tall *Nothofagus pumilio* forest. The lack of biomass‐GPP relationships in other regions may be due to shifting biomass allocation among leaf and stem components, thus rendering biomass a poor proxy for leaf area (Doležal et al. 2021). In particular, plants growing in dense assemblages tend to increase stem biomass to compete for light (Poorter et al. 2012).

An important consideration in our study is whether the results observed across a spatial gradient can be reliably extrapolated to temporal trajectories (Lovell et al. 2023). One challenge is that the changes in temperature—or associated variables such as the partial pressure of CO_2_—along these gradients do not necessarily represent future conditions. Another is that species responses, including range shifts and trait plasticity, often lag behind changes in temperature (Alexander et al. 2018; Mallen‐Cooper et al. 2023). There are many potential drivers of these lags, including dispersal limitation, biotic interactions, non‐temperature climate variables, and climatic variability (Jackson et al. 2009; Lawlor et al. 2024; Lovell et al. 2023). If there are lags in higher‐elevation species going extinct and in lower‐elevation species establishing upslope (i.e., poor climate tracking), it may be that many of our plots contain an unrealistic overabundance of lower‐elevation species and too few higher‐elevation species than will be present at that those temperatures. This would lead to an overestimation of GPP at a given temperature, since leaf area is typically higher in lower‐elevation plants (Luo et al. 2004). Another source of uncertainty in space‐for‐time studies is the potential for novel species interactions (Alexander et al. 2015). For example, upward‐shifting herbivores could accelerate the expansion of lower‐elevation plants, which tend to be less palatable than alpine plants (Descombes et al. 2020). In this case, we may have underestimated GPP at a given temperature. Nevertheless, until more data become available on species lags and interactions, and until DGVMs can accurately represent individual‐based carbon dynamics across all biomes, space‐for‐time substitutions will remain a valuable tool for estimating future GPP.

As the world warms, our findings imply differing trajectories for trees and ground‐layer plants in mountain ecosystems. Trees are likely to respond to macroclimate and, in the absence of disturbance and other recruitment limitations (e.g., dispersal, unfavourable microclimate), will probably expand into tundra and substantially increase ecosystem carbon uptake, with a concomitant but poorly understood effect on soil carbon decomposition (Parker et al. 2021). However, ground‐layer plants are likely to respond more to microclimate, and shifts in their ranges and functioning are likely to appear quite idiosyncratic, depending strongly on local context such as tree cover and cold air pooling. A key conclusion emerging from our results is that microclimate was poorly predicted by elevation at the global scale, yet explained more variation than did elevation in the carbon uptake of ground‐layer plants. It may even be that many counterintuitive responses and lagging range shifts along macroclimatic gradients (e.g., Auld et al. 2022; Mamantov et al. 2021) could be explained by a mismatch between the macro‐and microclimatic changes with elevation or latitude. As we aim to advance predictions of global change responses, our findings highlight the need to assess multiple scales of variability from microclimatic patches to entire continents.

## Author Contributions

**Max Mallen‐Cooper:** conceptualization, data curation, formal analysis, investigation, methodology, project administration, resources, visualization, writing – original draft, writing – review and editing. **Maja K. Sundqvist:** conceptualization, investigation, methodology, supervision, writing – review and editing. **David A. Wardle:** conceptualization, investigation, methodology, supervision, writing – review and editing. **Radim Šarlej:** investigation, methodology, writing – review and editing. **Rose E. Brinkhoff:** investigation, methodology, writing – review and editing. **Aimée T. Classen:** conceptualization, methodology, project administration, supervision, writing – review and editing. **Eliška Kuťáková:** investigation, writing – review and editing. **Daniel B. Metcalfe:** methodology, writing – review and editing. **M. Noelia Barrios‐Garcia:** project administration, writing – review and editing. **Julie R. Deslippe:** project administration, writing – review and editing. **Kobayashi Makoto:** project administration, writing – review and editing. **Jane Mallen‐Cooper:** investigation, methodology, project administration, writing – review and editing. **Barryette Oberholzer:** investigation, methodology, writing – review and editing. **Juan Paritsis:** investigation, project administration, writing – review and editing. **Jérémy Puissant:** project administration, writing – review and editing. **Mariano A. Rodriguez‐Cabal:** project administration, writing – review and editing. **Kohsuke Tanigawa:** investigation, methodology, writing – review and editing. **Susanna E. Venn:** investigation, project administration, writing – review and editing. **Paul Kardol:** conceptualization, funding acquisition, investigation, methodology, project administration, supervision, writing – review and editing.

## Funding

This work was supported by the European Research Council (864287‐THRESHOLD‐ERC‐2019‐COG) and U.S. Department of Energy, Office of Science, Office of Biological and Environmental Research, Terrestrial Ecosystem Sciences Program, DE‐FOA‐0002392.

## Conflicts of Interest

The authors declare no conflicts of interest.

## Supporting information


**Appendix S1:** gcb70877‐sup‐0001‐AppendixS1.pdf.

## Data Availability

All data and code used to produce the results of this study are publicly available on the Open Science Framework via the following link: https://doi.org/10.17605/OSF.IO/2NJZR.
