# Supplementary material for: Decoupled Climatic Drivers of Tree and Ground‐Layer Carbon Uptake in Mountain Ecosystems Around the World
Source: Glob Chang Biol. 2026 Apr 17;32:e70877. doi: 10.1111/gcb.70877 (PMC13090751; doi:10.1111/gcb.70877)
Supplement: Supplementary file 1 — Appendix S1: gcb70877‐sup‐0001‐AppendixS1.pdf. [file GCB-32-e70877-s001.pdf]

Supporting information.

Appendix 1. Additional information about study sites.

Australian Alps, Australia

The two northern transects in the Australian Alps (Thredbo, Stilwell) receive an average of 1800 mm of precipitation annually, while the southern transect (Hotham) receives about 1500 mm per year, although annual precipitation at both sites ranges from 1000–2700 mm (BOM, 2024). The snow pack in the alpine tundra and subalpine forests of the Australian Alps is usually 0.5–1.2 m deep at its peak, and lasts for 1.5–4.5 months, largely depending on aspect and elevation (Sanecki et al., 2006). In the higher alpine zone, the vegetation in our plots was typical of a tall alpine herbfield, largely dominated by species of *Poa* and *Celmisia*. In the subalpine Snow Gum (*Eucalyptus pauciflora* subsp. *niphophila*) forest and just above the treeline, the ground-layer vegetation was typically heath, dominated by *Oxylobium ellipticum*, *Nematolepis ovatifolia,* and *Kunzea muelleri*. Livestock grazing was a major ecological disturbance in the Australian Alps from the 1830s up until it was phased out in the northern part (Snowy Mountains) by 1972, and in the southern part (Victorian high country) by 2005 (Lockwood et al., 2014). While two native herbivores—the broad-toothed rat (*Mastacomys fuscus*) and the common wombat (*Vombatus ursinus*)—are known to graze at high elevations, their contribution to grazing activity is relatively minor compared to proliferating populations of exotic herbivores, namely horses, deer, hares and rabbits (Hartley et al., 2022).

Scandes Mountains, Sweden

In northern Sweden, we placed three transects within 25 km of the town of Abisko. The mean annual precipitation recorded in Abisko is 310 mm yr^{-1}, and snow depth tends to peak in late February at about 0.5 m, although wind-exposed sites generally have a much reduced snow

cover (Kohler et al., 2006). In this region, patches of high soil nutrients and high moisture generally support meadow vegetation communities, dominated by tall grasses and forbs, while drier patches with relatively lower soil nutrients support heath communities dominated by dwarf shrubs such as *Empetrum nigrum* subsp. *hermaphroditum* and *Betula nana* (Sundqvist et al., 2014). To avoid temperature effects being confounded by nutrient effects and hydrology, we selected plots with a ground-layer of heath vegetation, with the exception of the three lowest-elevation plots in the Latnjajaure transect, where only meadow vegetation was present. A distinct feature of elevation gradients in Sweden is that within these community types, there is very little species turnover in the ground-layer vegetation. The only tree species in our plots was mountain birch (*Betula pubescens* subsp. *czerepanovii*). Dung and shed antlers indicate that reindeer and moose were present at all transect sites, but visible signs of grazing were minimal. It is also important to note that there was an outbreak of winter moth larvae (*Operophtera brumata*) during the sampling year, which defoliated much of the mountain birch forest at the Suorooaivi transect and appeared to, directly or indirectly, negatively affect the underlying *Empetrum* shrubs.

<u>Rocky Mountains, USA</u>

We established three transects on mountain slopes near the Rocky Mountain Biological Laboratory in Gothic, Colorado, which receives an average of 605 mm of precipitation annually (for the period from 1994-2023; data from ncdc.noaa.gov). A distinct feature of the USA transects was that they were substantially higher in elevation than other countries (all plots >2900 m. a. s. l.; Table S1). In addition, the alpine tundra in this region occurs on particularly steep slopes (typically >30°) and has a soil surface covered in large shale pebbles (3-10 cm). Two conifer species dominate the subalpine forest: Engelmann spruce (*Picea engelmannii*) and subalpine fir (*Abies lasiocarpa*). Above the treeline, ground-layer

vegetation was dominated by a diverse mix of forbs (e.g., *Geum rossii*, *Senecio crassulus*), graminoids (e.g., *Deschampsia cespitosa*, *Carex scirpoidea*), and the dwarf shrub *Vaccinium cespitosum*. Below the treeline, the ground-layer was largely composed of forbs such as *Mertensia cilliata* and *Osmorhiza depauperata*, the dwarf shrub *Vaccinium myrtillus*, and the shrub *Ribes montigenum*. Cattle are able to graze close to our transects for a limited time during summer, but only two plots had visible signs of grazing. There was also some visible soil disturbance from digging vertebrates (e.g., yellow-bellied marmot, *Marmota flaviventris*), but it was largely restricted to the 50 m elevational band just above the treeline.

French Alps, France

We sampled three transects across the French Alps, near the towns of Chamonix (Plan de l'Aiguille transect), La Danchère (Réserve Intégrale du Lauvitel transect), and Névache, with nearby weather stations reporting a mean annual precipitation of 1280, 990 and 700 mm yr^{-1} respectively (data from meteofrance.com). Subalpine tree species varied among transects (Table S1) but the most common were European larch (*Larix decidua*) and Norway spruce (*Picea abies*). The ground-layer vegetation was typically a highly diverse mix of meadow flora and patchy shrubs such as the alpenrose (*Rhododendron ferrugineum*) and bilberry (*Vaccinium myrtillus*). The plots of one transect (Névache) were exposed to passing summer grazing (transhumance) by livestock, although areas of visible livestock disturbance were avoided, while the other transects were only exposed to wild grazers such as the chamois (*Rupicapra rupicapra*).

Patagonian Andes, Argentina

We established three transects in the Patagonian Andes surrounding the town of San Carlos de Bariloche in southern Argentina. There was a strong rainfall gradient across the transect sites,

from about 1790 mm yr^{-1} at Lopez in the west to 1200 mm yr^{-1} at Catedral in the east (data from mineria.gob.ar). A large proportion (65 %) of rainfall in the Bariloche region falls between May and August (period from 1991-2020; data from smn.gob.ar), typically producing humid snowy winters and dry summers. However, precipitation can be highly variable among years, due in large part to the El Niño-Southern Oscillation (Garbarini et al., 2016). We sampled during El Niño conditions, which led to a deeper snowpack and a later snowmelt in the mountains around Bariloche. In all our treed plots in this region, lenga beech (*Nothofagus pumilio*) was the sole tree species. Notably, the growth form of lenga beech transitions from tall trees at lower elevations to a dense zone of krummholz shrubs at the "treeline". The krummholz zone of lenga beech is known to be a hotspot of nutrient stocks and turnover (Frangi et al., 2005), and was characterised by minimal, or absent, ground-layer plant cover, likely due to high levels of shading. The vegetation in the alpine tundra zone was quite sparse (often < 50 % cover), and tended to form patches within the broader matrix of loose mineral soil. These patches were typically dominated by the cushion plant *Oreopolus glacialis*, *Poa* spp., and several dwarf shrubs, such as *Ochetophila nana* and *Azorella prolifera*. In the tall lenga beech forest, the ground-layer vegetation was dominated by shrubs such as *Berberis serro-dentata* and *Maytenus disticha*. We observed no signs of grazing or soil disturbance at any of the transect sites.

Literature cited

Frangi, J. L., Barrera, M. D., Richter, L. L., & Lugo, A. E. (2005). Nutrient cycling in Nothofagus pumilio forests along an altitudinal gradient in Tierra del Fuego, Argentina. *Forest Ecology and Management*, *217*(1), 80–94. https://doi.org/10.1016/j.foreco.2005.05.051

Garbarini, E., Skansi, M. M., Gonzalez, M. H., & Rolla, A. (2016). ENSO influence over precipitation in Argentina. In J. A. Daniels (Ed.), *Advances in Environmental Research* (Vol. 52, pp. 222–246). Nova Science Publishers.

Hartley, R., Blanchard, W., Schroder, M., Lindenmayer, D. B., Sato, C., & Scheele, B. C. (2022). Exotic herbivores dominate Australian high-elevation grasslands. *Conservation Science and Practice*, *4*(2), e601. https://doi.org/10.1111/csp2.601

Kohler, J., Brandt, O., Johansson, M., & Callaghan, T. (2006). A long-term Arctic snow depth record from Abisko, northern Sweden, 1913–2004. *Polar Research*, *25*(2), 91–113. https://doi.org/10.3402/polar.v25i2.6240

Lockwood, M., Mitchell, M., Moore, S. A., & Clement, S. (2014). Biodiversity governance and social-ecological system dynamics: Transformation in the Australian Alps. *Ecology and Society*, *19*(2), art13. https://doi.org/10.5751/ES-06393-190213

Sanecki, G. M., Green, K., Wood, H., & Lindenmayer, D. (2006). The Characteristics and Classification of Australian Snow Cover: An Ecological Perspective. *Arctic, Antarctic, and Alpine Research*, *38*(3), 429–435. https://doi.org/10.1657/1523-0430(2006)38[429:TCACOA]2.0.CO;2

Sundqvist, M. K., Wardle, D. A., Vincent, A., & Giesler, R. (2014). Contrasting nitrogen and phosphorus dynamics across an elevational gradient for subarctic tundra heath and meadow vegetation. *Plant and Soil*, *383*(1–2), 387–399. https://doi.org/10.1007/s11104-014-2179-5

Table S1. Key features of study sites.

Country	Mountain range	Sampling month(s)	No.# tundra plots	No.# forest plots	Tree species	Transect sites	Highest plot (m.a.s.l.)	Total elevation range (m)	Elevation of forest-tundra edge (m.a.s.l.)	Mean aspect (°)
Argentina	Patagonian Andes	Dec 2023	5	5	*Nothofagus pumilio*	Bellavista*<br>Catedral<br>Lopez	1763<br>1841<br>1750	176<br>262<br>223	1661<br>1700<br>1680	354 (N)<br>327 (NW)<br>317 (NW)
Australia	Australian Alps	Jan-Feb 2023	6	4	*Eucalyptus pauciflora* subsp. *niphophila*	Hotham<br>Stilwell<br>Thredbo	1854<br>2047<br>2080	120<br>172<br>221	1785<br>1947<br>1936	197 (S)<br>27 (NE)<br>146 (SE)
France	French Alps	Jul 2024	6	4	*Larix decidua, Pinus cembra, Picea abies, Aria edulis, Sorbus aucuparia*	Aiguille<br>Nevache<br>Lauvitel	2400<br>2248<br>2220	263<br>393<br>738	2077<br>2206<br>1579	330 (NW)<br>6 (N)<br>14 (N)
Sweden	Scandes	Jul 2023	7	3	*Betula pubescens* subsp. *czerepanovii*	Kaisepakte<br>Latnjajaure<br>Suorooaivi	1013<br>1240<br>938	598<br>562<br>553	521<br>810<br>522	72 (E)<br>221 (SW)<br>55 (NE)
USA	Rocky Mountains	Aug 2023	5	5	*Picea engelmannii, Abies lasiocarpa*	Bellview<br>Cinnamon<br>Slate	3615<br>3685<br>3719	482<br>402<br>788	3374<br>3488<br>3325	119 (SE)<br>32 (NE)<br>219 (SW)

*ground-layer fluxes were not measured

Table S2. Mean Diameter at Breast Height (DBH), leaf Gross Primary Productivity (GPP), Leaf Area Index (LAI) and light interception (1 - light transmittance) of studied tree species, the latter two based on fitted values from a linear model (predicted for the mean DBH of the species).

Country	Tree species	Mean DBH (cm)	% light intercepted (at mean DBH)	Mean leaf GPP (µmol m^{-2} s^{-1})	LAI (at mean DBH)
Argentina	*Nothofagus pumilio*	42	72	13.9	2.4
Australia	*Eucalyptus pauciflora* subsp. *niphophila*	41	64	12.8	1.5
France	*Aria edulis*	62	83	11.9	2.0
	Larix decidua	56	74	10.4	1.8
	Picea abies	63	92	5.2	2.8
	Pinus cembra	63	90	9.0	3.7
	Sorbus aucuparia	53	72	6.6	1.3
Sweden	*Betula pubescens* subsp. *czerepanovii*	15	50	13.2	1.1
USA	*Abies lasiocarpa*	66	85	3.9	2.9
	Picea engelmannii	66	88	4.7	3.7

Table S3. Plant cover statistics by country, averaged across all plots and elevations.

Country	Mean forest canopy cover (%)	Mean forest ground-layer plant cover (%)	Mean tundra ground-layer plant cover (%)
Argentina	86	39	46
Australia	56	98	98
France	58	96	98
Sweden	46	98	99
USA	54	73	76

Table S4. Results of generalized additive models between Gross Primary Productivity (GPP) of ground-layer vegetation and elevation, with effective degrees of freedom (EDFs) and associated p-values (note that values in the 0.0001–0.005 range are presented as 0.00).

Predictor	Country	Transect	EDF	F-statistic	p-value
Elevation	Argentina	Catedral	0.07	0.02	0.10
		Lopez	0.28	0.36	**0.00**
	Australia	Hotham	0.53	0.57	**0.02**
		Stilwell	0.23	0.10	0.17
		Thredbo	0.29	0.03	0.54
	France	Aiguille	0.66	2.29	**<0.0001**
		Lauvitel	0.82	2.33	**0.00**
		Nevache	0.74	0.47	**0.05**
	Sweden	Kaisepakte	1.02	0.46	0.12
		Latnjajaure	1.01	0.87	**0.02**
		Suorooaivi	1.01	0.06	0.59
	USA	Bellview	1.00	0.77	**0.04**
		Cinnamon	1.00	1.31	**0.01**
		Slate	0.99	9.53	**<0.0001**
Control variable: air temperature during measurement	Argentina	Catedral	0.00	0.00	0.76
		Lopez	0.77	1.50	**0.02**
	Australia	Hotham	0.00	0.00	0.64
		Stilwell	0.00	0.00	0.32
		Thredbo	0.00	0.00	0.80
	France	Aiguille	0.08	0.05	0.28
		Lauvitel	0.96	6.05	**0.00**
		Nevache	0.72	1.23	**0.05**
	Sweden	Kaisepakte	0.00	0.00	0.60
		Latnjajaure	0.00	0.00	0.52
		Suorooaivi	0.00	0.00	0.57
	USA	Bellview	0.00	0.00	0.77
		Cinnamon	0.00	0.00	0.62
		Slate	0.34	0.26	0.22
Control variable: soil moisture during measurement	Argentina	Catedral	1.86	19.25	**<0.0001**
		Lopez	1.04	11.53	**<0.0001**
	Australia	Hotham	0.28	0.18	0.26
		Stilwell	0.00	0.00	0.31
		Thredbo	0.12	0.06	0.27
	France	Aiguille	0.00	0.00	0.60
		Lauvitel	0.00	0.00	0.26
		Nevache	0.81	2.04	**0.02**

Sweden	Kaisepakte	0.00	0.00	0.83
	Latnjajaure	0.24	0.16	0.25
	Suorooaivi	0.57	0.67	0.13
USA	Bellview	0.00	0.00	0.42
	Cinnamon	0.00	0.00	0.94
	Slate	0.00	0.00	0.64

Table S5. Results of generalized additive models between Gross Primary Productivity (GPP) and the annual number of growing degree days, with effective degrees of freedom (EDFs) and associated p-values (note that values in the 0.0001–0.0049 range are presented as 0.00).

Predictor	Country	Transect	EDF	F-statistic	p-value
Growing degree days	Argentina	Catedral	**2.01**	14.77	**<0.0001**
		Lopez	**2.14**	5.58	**<0.0001**
	Australia	Hotham	0.94	2.37	**0.00**
		Stilwell	0.00	0.00	0.61
		Thredbo	0.85	1.11	**0.01**
	France	Aiguille	0.97	1.24	**0.01**
		Lauvitel	0.67	0.38	0.07
		Nevache	0.00	0.00	0.43
	Sweden	Kaisepakte	1.86	2.03	**0.00**
		Latnjajaure	0.00	0.00	0.31
		Suorooaivi	0.87	1.11	**0.01**
	USA	Bellview	0.95	2.29	**0.00**
		Cinnamon	0.95	3.22	**<0.0001**
		Slate	0.40	0.12	0.19
Control variable: air temperature during measurement	Argentina	Catedral	0.78	1.33	**0.04**
		Lopez	0.00	0.00	0.78
	Australia	Hotham	0.00	0.00	0.62
		Stilwell	0.00	0.00	0.66
		Thredbo	0.00	0.00	0.80
	France	Aiguille	0.00	0.00	0.68
		Lauvitel	0.91	4.04	**0.00**
		Nevache	0.84	2.40	**0.02**
	Sweden	Kaisepakte	0.00	0.00	0.90
		Latnjajaure	0.00	0.00	0.60
		Suorooaivi	0.00	0.00	0.81
	USA	Bellview	0.00	0.00	0.58
		Cinnamon	0.00	0.00	0.75
		Slate	0.62	0.80	0.10
Control variable: soil moisture during measurement	Argentina	Catedral	0.00	0.00	0.39
		Lopez	0.42	0.35	0.15
	Australia	Hotham	0.00	0.00	0.45
		Stilwell	0.00	0.00	0.65
		Thredbo	0.00	0.00	0.48
	France	Aiguille	0.00	0.00	0.57
		Lauvitel	0.77	1.36	**0.03**
		Nevache	0.90	4.35	**0.00**
	Sweden	Kaisepakte	0.00	0.00	0.73
		Latnjajaure	0.60	0.76	0.11

		Suorooaivi	0.64	0.85	0.10
	USA	Bellview	0.00	0.00	0.38
		Cinnamon	0.00	0.00	0.33
		Slate	0.97	10.66	**<0.0001**

Table S6. Results of generalized additive models between Gross Primary Productivity (GPP) and mean growing season temperature (°C), with effective degrees of freedom (EDFs) and associated p-values (note that values in the 0.0001–0.005 range are presented as 0.00).

Predictor	Country	Transect	EDF	F-statistic	p-value
Mean growing season temperature at the soil surface	Argentina	Catedral	0.92	1.79	**0.00**
		Lopez	0.95	3.04	**<0.0001**
	Australia	Hotham	0.00	0.00	0.48
		Stilwell	0.00	0.00	0.76
		Thredbo	0.34	0.10	0.21
	France	Aiguille	1.00	2.13	**0.00**
		Lauvitel	0.00	0.00	0.91
		Nevache	0.00	0.00	0.58
	Sweden	Kaisepakte	0.00	0.00	0.84
		Latnjajaure	0.00	0.00	0.34
		Suorooaivi	0.00	0.00	0.44
	USA	Bellview	0.00	0.00	0.49
		Cinnamon	0.00	0.00	0.56
		Slate	0.76	0.56	0.05
Control variable: air temperature during measurement	Argentina	Catedral	0.00	0.00	0.69
		Lopez	0.00	0.00	0.96
	Australia	Hotham	0.86	2.62	**0.01**
		Stilwell	0.00	0.00	0.41
		Thredbo	0.00	0.00	0.90
	France	Aiguille	0.79	1.86	**0.03**
		Lauvitel	0.95	5.21	**0.00**
		Nevache	0.90	3.74	**0.00**
	Sweden	Kaisepakte	0.00	0.00	0.65
		Latnjajaure	0.00	0.00	0.76
		Suorooaivi	0.00	0.00	0.67
	USA	Bellview	0.00	0.00	0.81
		Cinnamon	0.00	0.00	0.89
		Slate	0.75	1.42	0.05
Control variable: soil moisture during measurement	Argentina	Catedral	1.68	30.31	**<0.0001**
		Lopez	0.99	18.20	**<0.0001**
	Australia	Hotham	0.88	2.82	**0.01**
		Stilwell	0.00	0.00	0.61
		Thredbo	0.42	0.34	0.19
	France	Aiguille	0.00	0.00	0.39
		Lauvitel	0.99	4.80	**0.00**
		Nevache	0.89	3.73	**0.00**
	Sweden	Kaisepakte	0.00	0.00	0.62
		Latnjajaure	0.82	2.20	**0.02**

		Suorooaivi	0.62	0.59	0.16
	USA	Bellview	0.84	2.58	**0.01**
		Cinnamon	0.90	4.06	**0.00**
		Slate	0.99	22.41	**<0.0001**

Table S7. Results of generalized additive models between Gross Primary Productivity (GPP) and mean gravimetric soil moisture during the growing season (%; converted from raw sensor data using calibrations of local soil), with effective degrees of freedom (EDFs) and associated p-values (note that values in the 0.0001–0.005 range are presented as 0.00).

Predictor	Country	Transect	EDF	F-statistic	p-value
Mean soil moisture during the growing season	Argentina	Catedral	1.35	9.29	**<0.0001**
		Lopez	**2.13**	4.79	**<0.0001**
	Australia	Hotham	0.92	1.52	**0.00**
		Stilwell	0.00	0.00	0.55
		Thredbo	0.00	0.00	0.78
	France	Aiguille	0.39	0.13	0.19
		Lauvitel	**2.08**	1.83	**0.00**
		Nevache	0.00	0.00	0.61
	Sweden	Kaisepakte	0.00	0.00	0.37
		Latnjajaure	0.00	0.00	0.30
		Suorooaivi	0.23	0.06	0.25
	USA	Bellview	0.00	0.00	0.67
		Cinnamon	0.94	2.42	**0.00**
		Slate	1.98	11.29	**<0.0001**
Control variable: air temperature during measurement	Argentina	Catedral	0.04	0.03	0.26
		Lopez	0.00	0.00	0.89
	Australia	Hotham	0.00	0.00	0.64
		Stilwell	0.00	0.00	0.49
		Thredbo	0.00	0.00	0.85
	France	Aiguille	0.32	0.24	0.21
		Lauvitel	0.00	0.00	0.55
		Nevache	0.87	3.17	0.01
	Sweden	Kaisepakte	0.00	0.00	0.84
		Latnjajaure	0.00	0.00	0.71
		Suorooaivi	0.00	0.00	0.65
	USA	Bellview	0.00	0.00	0.81
		Cinnamon	0.00	0.00	0.63
		Slate	0.72	1.26	0.06
Control variable: soil moisture during measurement	Argentina	Catedral	0.26	0.15	0.26
		Lopez	0.57	0.68	0.08
	Australia	Hotham	0.00	0.00	0.53
		Stilwell	0.00	0.00	0.84
		Thredbo	0.66	0.92	0.09
	France	Aiguille	0.70	1.02	0.08
		Lauvitel	0.17	0.10	0.28
		Nevache	0.89	3.69	0.00

Sweden	Kaisepakte	0.00	0.00	0.80
	Latnjajaure	0.76	1.56	0.04
	Suorooaivi	0.73	0.79	0.12
USA	Bellview	0.87	3.20	0.01
	Cinnamon	0.00	0.00	0.97
	Slate	0.00	0.00	0.19

Table S8. Results of generalized additive models between microclimate variables and elevation, with effective degrees of freedom (EDFs) and associated p-values (note that values in the 0.0001–0.005 range are presented as 0.00).

Microclimate variable	Country	Transect	EDF	F-statistic	P-value
Growing degree days	Argentina	Bellavista	1.01	3.44	**<0.0001**
		Catedral	1.58	2.22	**0.0001**
		Lopez	0.00	0.00	0.36
	Australia	Hotham	1.94	20.71	**<0.0001**
		Stilwell	1.98	20.12	**<0.0001**
		Thredbo	1.98	13.30	**<0.0001**
	France	Aiguille	1.16	2.78	**<0.0001**
		Lauvitel	1.42	7.80	**<0.0001**
		Nevache	1.07	3.04	**<0.0001**
	Sweden	Kaisepakte	1.02	11.18	**<0.0001**
		Latnjajaure	1.97	13.67	**<0.0001**
		Suorooaivi	1.72	7.67	**<0.0001**
	USA	Bellview	1.01	12.45	**<0.0001**
		Cinnamon	1.92	50.71	**<0.0001**
		Slate	1.02	7.60	**<0.0001**
Mean growing season temperature at the soil surface	Argentina	Bellavista	0.00	0.00	0.80
		Catedral	0.00	0.00	0.95
		Lopez	0.88	1.08	**0.01**
	Australia	Hotham	0.00	0.00	0.56
		Stilwell	1.00	0.43	0.08
		Thredbo	0.08	0.01	0.32
	France	Aiguille	0.85	0.71	**0.02**
		Lauvitel	1.11	1.70	**0.00**
		Nevache	1.79	3.77	**<0.0001**
	Sweden	Kaisepakte	0.41	0.12	0.19
		Latnjajaure	0.72	0.43	**0.06**
		Suorooaivi	0.29	0.07	0.23
	USA	Bellview	**2.06**	11.93	**<0.0001**
		Cinnamon	**2.02**	4.29	**<0.0001**
		Slate	0.99	4.51	**<0.0001**

Table S9. Results of cold air pooling analysis in the subalpine/subarctic forest zone (% of time in which a higher elevation plot had a higher air temperature, measured at 2 m above ground, than a lower elevation plot).

Country	Transect	% inverted ($T_{high} > T_{low}$)	% inverted during growing season	% inverted during snow period
Argentina	Bellavista	15.0	19.4	11.0
	Catedral	29.8	34.8	24.4
	Lopez	8.1	10.0	6.3
Australia	Hotham	18.1	18.9	16.9
	Stilwell	28.1	25.6	31.6
	Thredbo	15.8	16.9	14.2
France	Aiguille	28.6	46.1	17.3
	Lauvitel	5.1	5.2	5.1
	Nevache	21.7	21.0	22.1
Sweden	Kaisepakte	41.5	29.6	46.9
	Latnjajaure	52.0	47.8	53.8
	Suorooaivi	22.9	17.4	25.9
USA	Bellview	58.4	69.6	53.5
	Cinnamon	51.3	59.7	48.3
	Slate	20.7	22.0	20.2

Table S10. Results of generalized additive models between Gross Primary Productivity (GPP) and the community-weighted mean of leaf nitrogen per area (N_{area}), with effective degrees of freedom (EDFs) and associated p-values (note that values in the 0.0001–0.005 range are presented as 0.00).

Predictor	Country	Transect	EDF	F-statistic	p-value
Leaf N_{area}	Argentina	Catedral	0.00	0.00	0.38
		Lopez	0.18	0.04	0.29
	Australia	Hotham	0.00	0.00	0.48
		Stilwell	0.71	0.46	0.06
		Thredbo	0.67	0.40	0.08
	France	Aiguille	0.91	2.35	**0.00**
		Lauvitel	**2.12**	3.93	**<0.0001**
		Nevache	1.91	2.55	**0.00**
	Sweden	Kaisepakte	0.19	0.05	0.26
		Latnjajaure	0.00	0.00	0.87
		Suorooaivi	0.00	0.00	0.85
	USA	Bellview	0.89	1.59	**0.00**
		Cinnamon	0.00	0.00	0.34
		Slate	0.65	0.35	0.10
Control variable: air temperature during measurement	Argentina	Catedral	0.00	0.00	0.76
		Lopez	0.00	0.00	0.80
	Australia	Hotham	0.85	2.45	**0.01**
		Stilwell	0.28	0.20	0.22
		Thredbo	0.00	0.00	0.93
	France	Aiguille	0.00	0.00	0.60
		Lauvitel	0.11	0.06	0.28
		Nevache	0.00	0.00	0.43
	Sweden	Kaisepakte	0.00	0.00	0.62
		Latnjajaure	0.00	0.00	0.74
		Suorooaivi	0.00	0.00	0.65
	USA	Bellview	0.00	0.00	0.73
		Cinnamon	0.00	0.00	0.89
		Slate	0.76	1.54	**0.04**
Control variable: soil moisture	Argentina	Catedral	1.92	41.03	**<0.0001**
		Lopez	1.89	24.88	**<0.0001**
	Australia	Hotham	0.87	2.70	**0.01**
		Stilwell	0.24	0.16	0.22

during measurement		Thredbo	0.00	0.00	0.42
	France	Aiguille	0.00	0.00	0.81
		Lauvitel	0.00	0.00	0.35
		Nevache	0.15	0.08	0.27
	Sweden	Kaisepakte	0.00	0.00	0.60
		Latnjajaure	0.85	2.66	**0.01**
		Suorooaivi	0.65	0.66	0.14
	USA	Bellview	0.00	0.00	0.68
		Cinnamon	0.90	3.80	**0.00**
		Slate	0.98	19.45	**<0.0001**

Table S11. Results of generalized additive models between Gross Primary Productivity (GPP) and aboveground ground-layer biomass (kg m^{-2}), with effective degrees of freedom (EDFs) and associated p-values (note that values in the 0.0001–0.005 range are presented as 0.00).

Predictor	Country	Transect	EDF	F-statistic	p-value
Biomass	Argentina	Catedral	1.00	8.35	**<0.0001**
		Lopez	1.07	5.73	**<0.0001**
	Australia	Hotham	0.00	0.00	0.75
		Stilwell	0.00	0.00	0.43
		Thredbo	0.75	0.60	**0.05**
	France	Aiguille	0.73	0.52	0.06
		Lauvitel	0.00	0.00	0.44
		Nevache	0.00	0.00	0.77
	Sweden	Kaisepakte	0.00	0.00	0.34
		Latnjajaure	0.00	0.00	0.40
		Suorooaivi	0.00	0.00	0.37
	USA	Bellview	0.91	2.36	**0.00**
		Cinnamon	0.94	3.61	**0.00**
		Slate	0.99	17.34	**<0.0001**
Control variable: air temperature during measurement	Argentina	Catedral	0.00	0.00	0.48
		Lopez	0.00	0.00	0.36
	Australia	Hotham	0.91	3.86	**0.00**
		Stilwell	0.00	0.00	0.54
		Thredbo	0.00	0.00	0.95
	France	Aiguille	0.14	0.09	0.26
		Lauvitel	0.93	4.32	**0.00**
		Nevache	0.86	2.74	**0.01**
	Sweden	Kaisepakte	0.00	0.00	0.9
		Latnjajaure	0.00	0.00	0.71
		Suorooaivi	0.00	0.00	0.59
	USA	Bellview	0.00	0.00	0.96
		Cinnamon	0.00	0.00	0.49
		Slate	0.87	3.03	**0.01**
Control variable: soil moisture during measurement	Argentina	Catedral	0.96	9.40	**<0.0001**
		Lopez	0.95	7.09	**0.00**
	Australia	Hotham	0.93	3.94	**0.00**
		Stilwell	0.00	0.00	0.98
		Thredbo	0.00	0.00	0.32
	France	Aiguille	0.83	1.81	**0.03**
		Lauvitel	0.98	5.02	**0.00**
		Nevache	0.89	3.71	**0.00**
	Sweden	Kaisepakte	0.00	0.00	0.90
		Latnjajaure	0.72	1.26	0.06

	Suorooaivi	0.95	1.05	0.12
USA	Bellview	0.00	0.00	0.79
	Cinnamon	0.00	0.00	0.91
	Slate	0.00	0.00	0.95

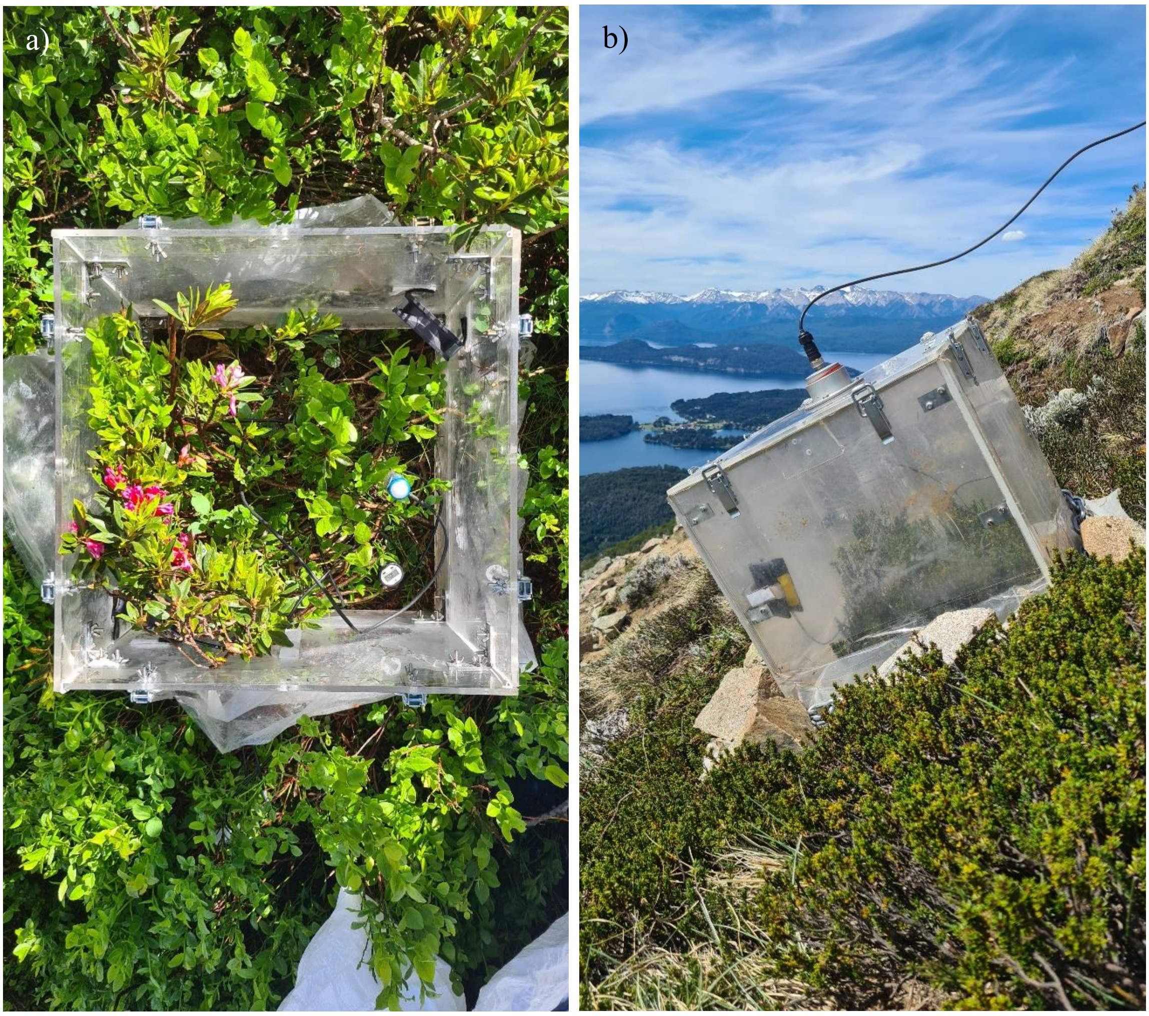

Figure S1. Ground-layer flux chamber, with a) view from above with lid off, and (b) view from side with lid on. Photos taken by Max Mallen-Cooper at the Nevache (France) and Lopez (Argentina) transects, respectively.

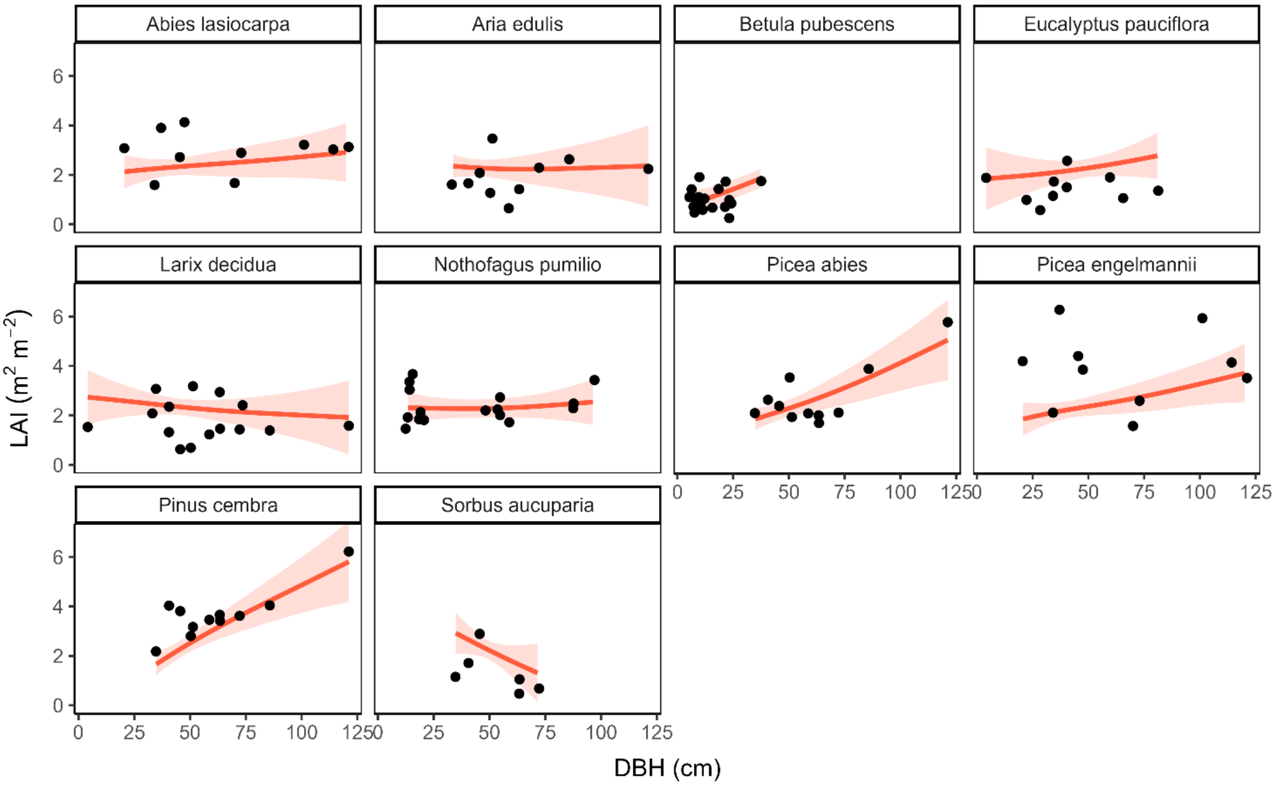

Figure S2. Generalised additive model (GAM; adjusted R^2 = 0.36) of the relationship between Leaf Area Index (LAI) and diameter at breast height (DBH) in 'calibration trees' of each species, with a 95 % confidence interval ribbon around mean predictions and raw data shown as points.

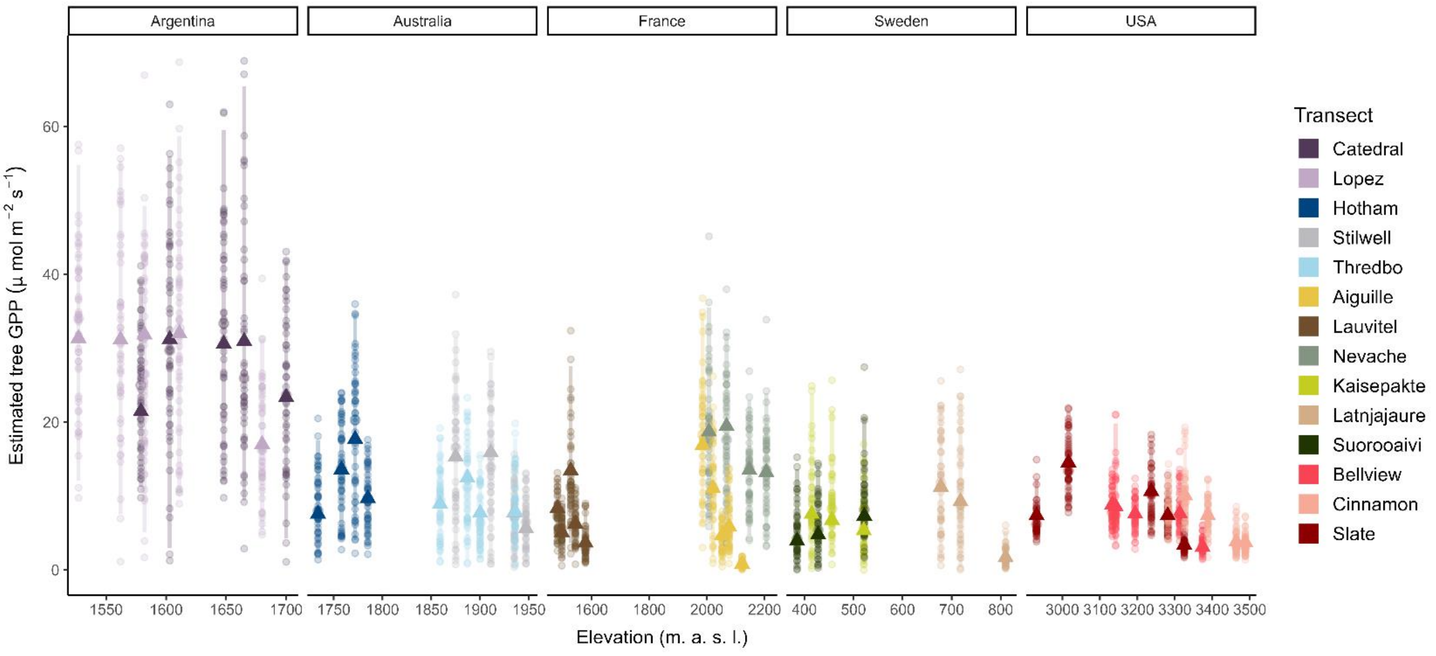

Figure S3. Results of error propagation analysis showing mean estimated tree GPP in each plot (triangles) and simulated resamples (circles) drawing from the variation in LAI-DBH relationships and leaf-level fluxes. The vertical line indicates the 95 % confidence interval of all resamples, and colours represent different transects.

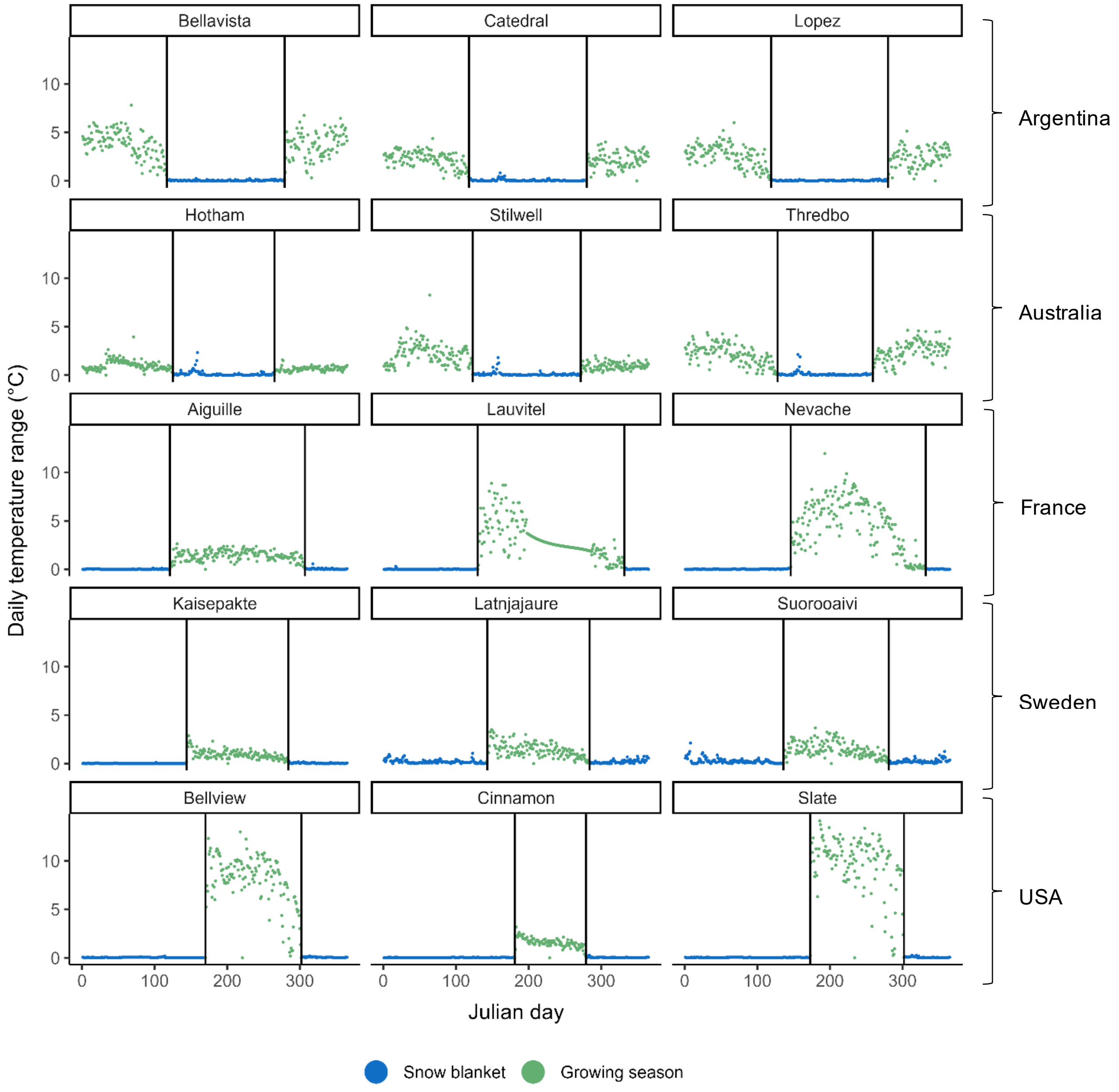

Figure S4. Growing season cut-offs using a 0.3 °C daily temperature range criterion (measured at the soil surface), with one example plot shown from each transect.

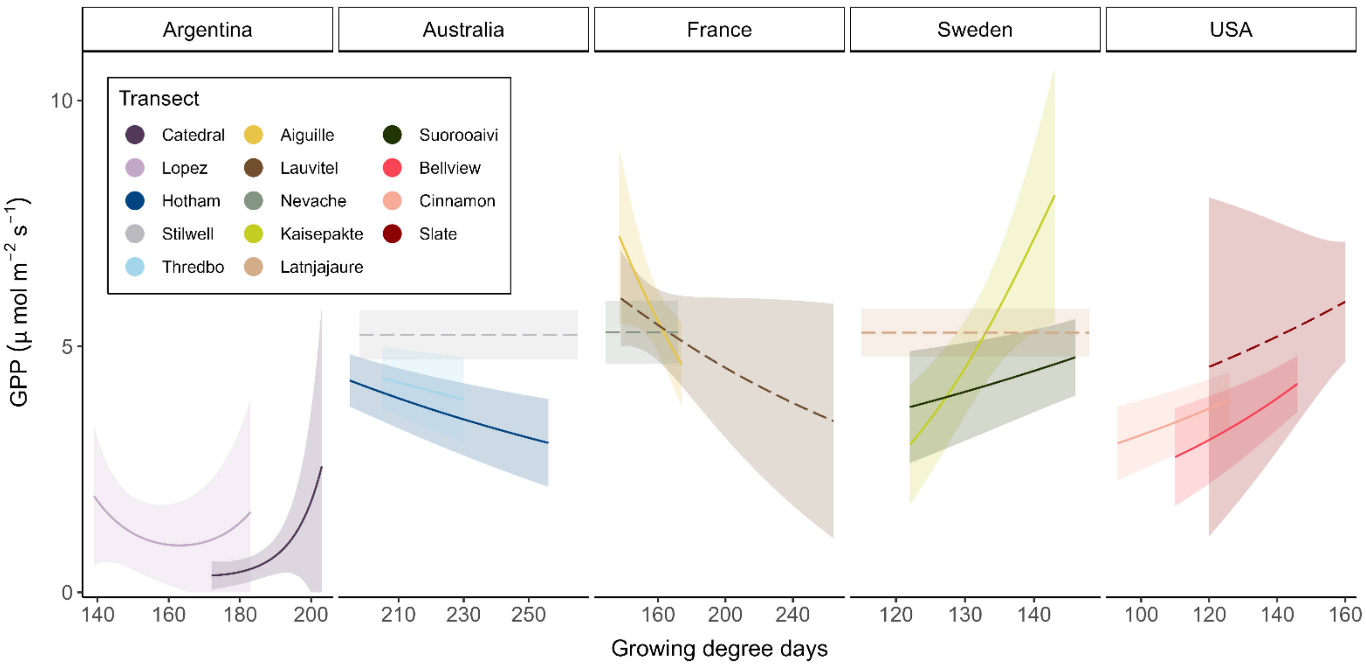

Figure S5. Modelled relationships (± 95 % CI) between ground-layer vegetation Gross Primary Productivity (GPP, standardised to 600 PAR open-sky conditions) and the annual number of growing degree days (>5 °C maximum temperature at ground level) across forest-tundra gradients, plotted at a standard measurement temperature of 25 °C. Significant relationships (at $p < 0.05$; see Table S7 for statistical results) are shown as a solid line, non-significant relationships are shown as dashed lines, and different colours represent different transects.

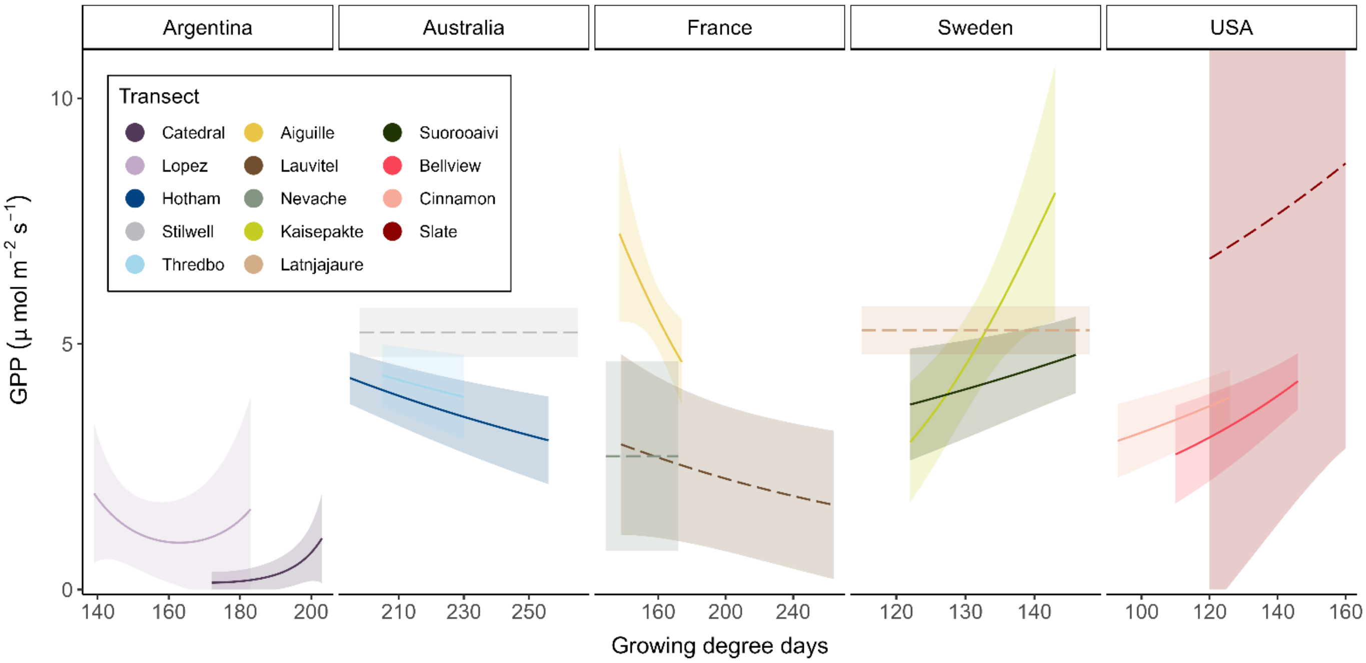

Figure S6. Modelled relationships (± 95 % CI) between ground-layer vegetation Gross Primary Productivity (GPP, standardised to 600 PAR open-sky conditions) and the annual number of growing degree days (>5 °C maximum temperature at ground level) across forest-tundra gradients, plotted at a standard measurement temperature of 10 °C. Significant relationships (at $p < 0.05$; see Table S7 for statistical results) are shown as a solid line, non-significant relationships are shown as dashed lines, and different colours represent different transects.

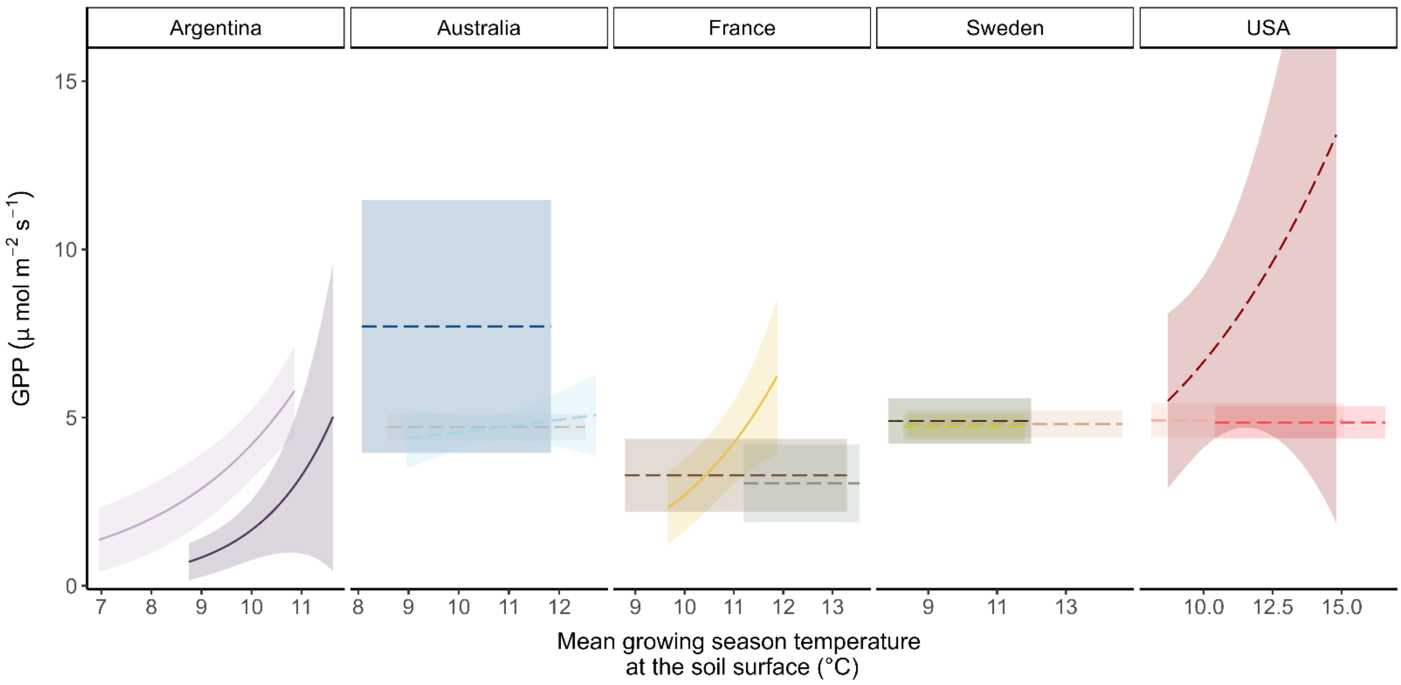

Figure S7. Modelled relationships (± 95 % CI) between ground-layer vegetation Gross Primary Productivity (GPP, s tandardised to 600 PAR open-sky conditions) and the mean microclimate temperature measured at the soil surface during the growing season. Significant relationships are shown as a solid line, with non-significant relationships shown as dashed lines, and different colours represent different transects. Raw plot-level data is not shown since it does not reflect the standardised conditions used to compare GPP among transects (18 °C air temperature and 50 % soil moisture; see Methods).

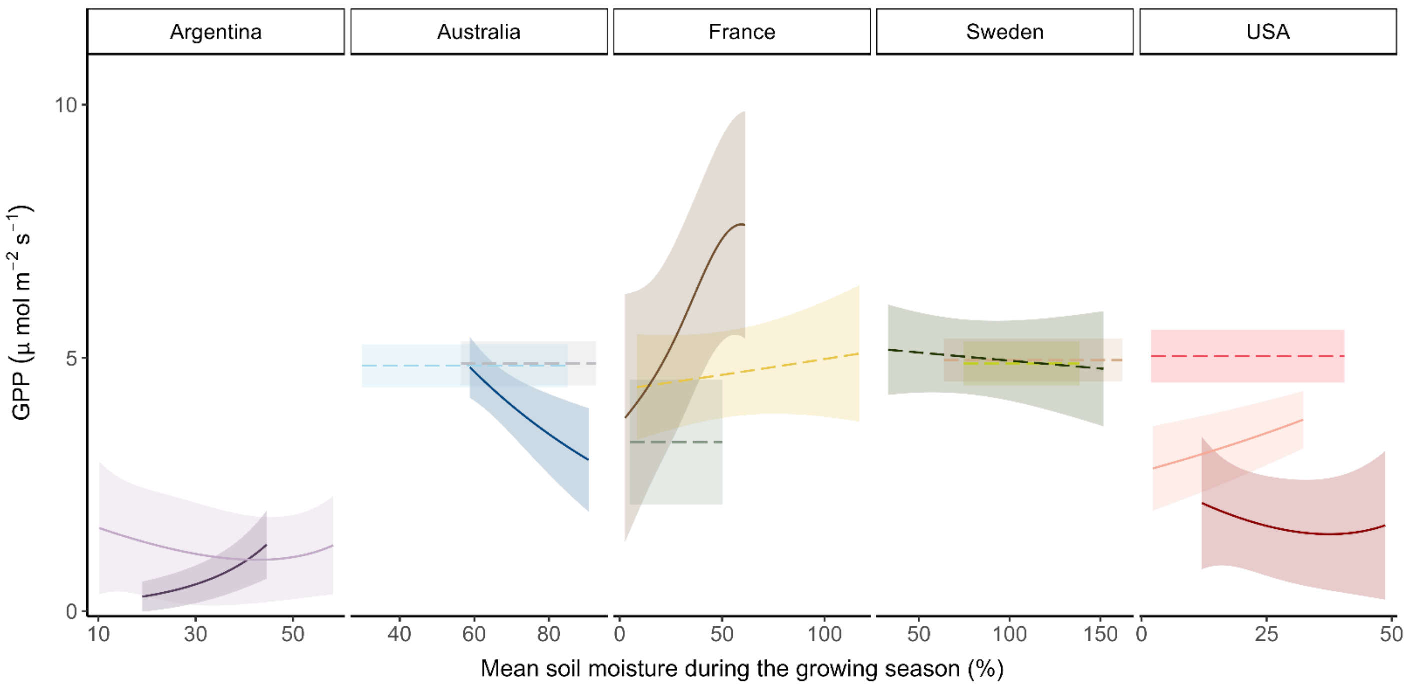

Figure S8. Modelled relationships (± 95 % CI) between ground-layer vegetation Gross Primary Productivity (GPP, standardised to 600 PAR open-sky conditions) and mean gravimetric soil moisture content during the growing season (converted from raw sensor data using calibrations of local soil). Significant relationships are shown as a solid line, with non-significant relationships shown as dashed lines, and different colours represent different transects. Raw plot-level data is not shown since it does not reflect the standardised conditions used to compare GPP among transects (18 °C air temperature and 50 % soil moisture; see Methods).

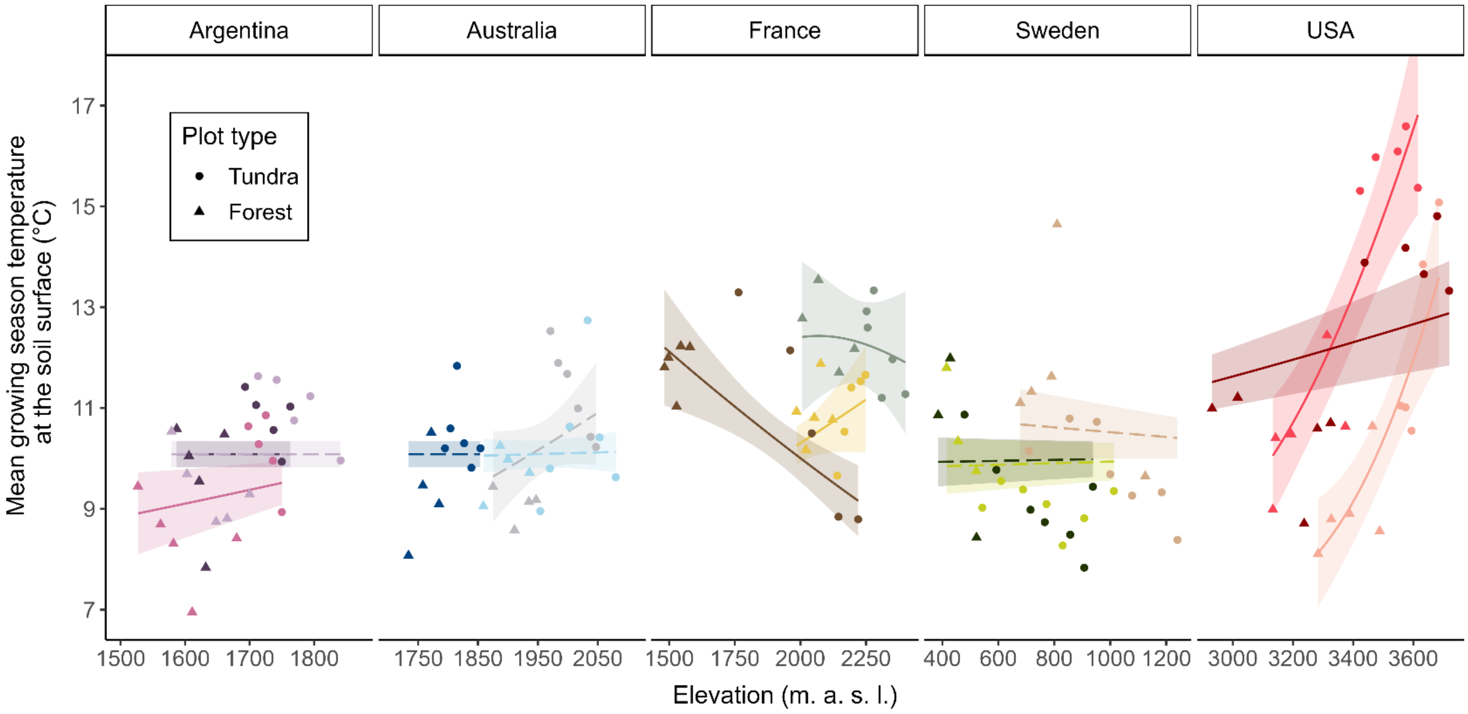

Figure S9. Modelled relationships (± 95 % CI) between mean microclimate temperature during the growing season and elevation. Significant relationships (at $p < 0.05$, based on approximate Wald-type tests for Generalised Additive Models; see Table S2 for statistical results) are shown as a solid line, with non-significant relationships shown as dashed lines, and different colours represent different transects.

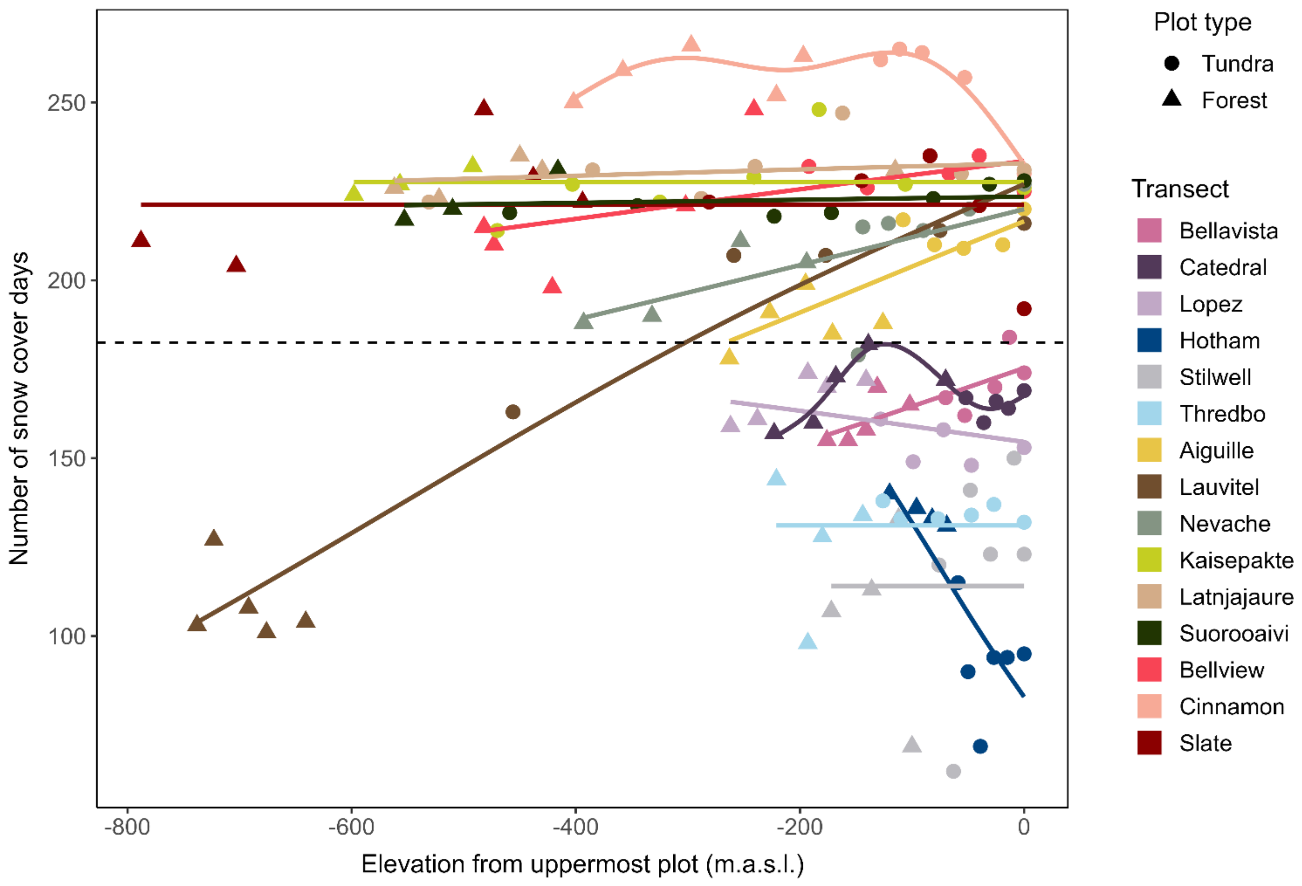

Figure S10. Relationship between elevation (relative to the uppermost plot) and the annual number of snow cover days, with points representing plots, solid lines representing fitted GAMs, and the dotted line showing six months.
